# MicroRNA miR-378-3p is a novel regulator of endothelial autophagy and function

**DOI:** 10.1016/j.jmccpl.2022.100027

**Published:** 2022-12-08

**Authors:** Shuhan Bu, Jameela J. Joseph, Hien C. Nguyen, Mehroz Ehsan, Berk Rasheed, Aman Singh, Mohammad Qadura, Jefferson C. Frisbee, Krishna K. Singh

**Affiliations:** a Department of Medical Biophysics, Schulich School of Medicine and Dentistry, University of Western Ontario, London, ON N6A 5C1, Canada; b Department of Anatomy and Cell Biology, Schulich School of Medicine and Dentistry, University of Western Ontario, London, ON N6A 5C1, Canada; c Department of Pathology and Laboratory Medicine, Schulich School of Medicine and Dentistry, University of Western Ontario, London, ON N6A 5C1, Canada; d Institute of Medical Science, University of Toronto, Toronto, ON M5S 1A1, Canada

**Keywords:** MicroRNA (miRNA), miR-378-3p, Endothelial cell, Autophagy, Autophagy-related protein 7 (ATG7), Endothelial function, Nitric oxide synthase

## Abstract

Autophagy is a highly conserved cellular process in which cytoplasmic materials are internalized into an autophagosome that later fuses with a lysosome for their degradation and recycling. MicroRNAs (miRNAs) are integral regulators in various cellular processes including autophagy and endothelial function. Accordingly, we hypothesize that miRNA, miR-378-3p, is an essential regulator of endothelial autophagy and endothelial function. MiR-378-3p expression was measured following inhibition and activation of autophagy in endothelial cells. A gain- or loss-of function approach was employed to either overexpress or inhibit the expression of miR-378-3p, respectively, in cultured endothelial cells, and markers of autophagy and indices of endothelial function, such as proliferation, migration and tube forming potential were measured. Inhibition and activation of autophagy up- and down-regulated the expression of miR-378-3p, respectively. Furthermore, miR-378a-3p overexpression was associated with impaired autophagy indicated by a reduced LC3-II/LC3-I ratio, and endothelial function indicated by increased proliferation associated with reduced p21 expression, reduced angiogenic potential and increased migration, which were associated with reduced expression of endothelial nitric oxide synthase (eNOS), an essential regulator of endothelial function. Accordingly, miR-378a-3p inhibition was associated with reduced cell proliferation, migration and increased eNOS in endothelial cells. Apoptosis was not affected in cells transfected with antagomir. Using *in silico* approach, *Protein Disulfide Isomerase Family A Member 4* (PDIA-4) was identified and confirmed as a target of miR-378-3p. PDIA-4 expression was significantly reduced or enhanced in miR-378-3p-overexpressing or -silenced endothelial cells, respectively. Our findings show an inverse relationship between miR-378-3p and endothelial autophagy and function, providing a novel insight about the epigenetic regulation of these processes.

## Highlights

• MicroRNA miR-378-3p expression inversely correlates with endothelial autophagy.
• MiR-378-3p expression inversely regulates endothelial function.
• MiR-378-3p negatively regulates endothelial nitric oxide synthase expression.
• MiR-378-3p targets *PDIA-4* gene in endothelial cells.

## Graphical abstract

**Figure:**
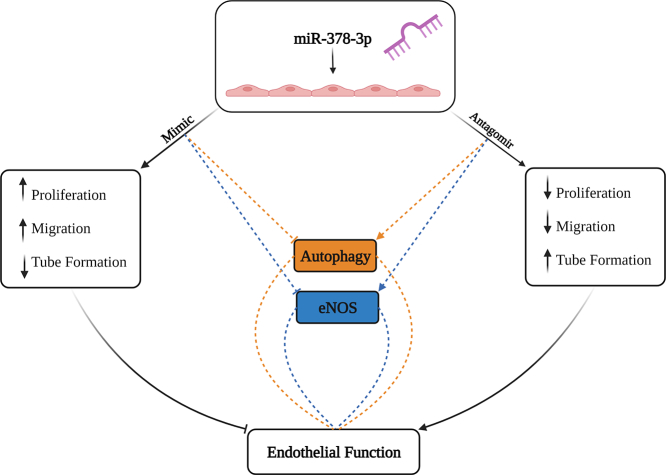
MiR-378-3p regulates endothelial function and autophagy (Created with BioRender.com).

## 1 Introduction

Endothelial cells (ECs) form a single cell layer lining blood vasculature and have critical functions, such as regulating nutrient exchanges between blood and tissues, the inflammatory responses, wound healing, and helping maintain vascular tone [1]. ECs conduct autophagy, which serves the critical role of maintaining homeostasis. Imbalances in autophagic influx could lead to endothelial dysfunction, which is linked to various cardiovascular diseases (CVDs) such as atherosclerosis [2], [3]. Macroautophagy or autophagy is a highly conserved pathway that maintains cellular homeostasis [3]. In this process, a cell turns over unnecessary proteins and organelles as needed for constituents that are better used to sustain the cell's survival [4]. The turned over cellular components are internalized into the double membraned autophagosomes in the cytoplasm, which ultimately fuses with lysosomes for digestion into basic materials such as amino acids [5]. Autophagy is usually activated as a pro-survival response to various stress signals derived from nutrient deprivation and other cellular injuries scenarios [6]. The formation of the autophagosomes involves two ubiquitin-like conjugation systems. One of these systems is the autophagy related gene 5 (ATG5)-ATG12 conjugation system formed by the protein ATG5 conjugating to ATG12 with the help of ubiquitin-like enzymes ATG7 and ATG10, promoting the elongation of the autophagosomal membrane. The second conjugation system involves microtubule-associated protein 1 light chain 3 (LC3), which is cleaved by the protease ATG4B and transformed to its mature form, LC3-I, which conjugates with phosphatidylethanolamine (PE) to form the lipidated version, LC3-II [5]. The ratio of LC3-II/LC3I has thus become a common method to measure autophagic activity in cells [7]. P62 protein is another marker of autophagy as it is associated with cargo proteins and gets degraded in the process of autophagy. Autophagy inhibition, therefore, leads to accumulation, therefore, increased accumulation of P62 [8]. ATG7 is a central regulator of endothelial autophagy as knocking down ATG7 leads to impaired autophagic influx and inhibition of autophagy. Dysregulation of autophagy is involved in a wide range of diseases, including neurodegenerative diseases, CVDs and cancer [5].

MicroRNAs (miRNAs) are non-coding RNAs that can target mRNAs leading to mRNA’ degradation, translational repression or a complete block of translation [3]. MiRNAs play essential roles in various biological processes including cell development and differentiation [9]. MicroRNAs have been increasingly investigated for their roles in autophagy and endothelial functions [3]. A study found that the miR-100 has an anti-inflammatory role in vascular responses to injury by stimulating endothelial autophagy [10]. Moreover, the miR-378-3p was found to be transcribed from the gene encoding for the metabolic regulator PGC-1β and is involved in angiogenesis, muscle differentiation and cell metabolism [9]. MiR-378-3p was also shown to play a crucial role in regulating proliferation and differentiation of myoblasts [11], as well as promoting vascularization of skeletal muscle following ischaemic injury in mice [12]. Li *et al* showed the potential of miR-378-3p to act as a tumor suppressor in colorectal cancer [13]. A recent study showed the role of miR-378-3p in regulating autophagy and apoptosis in mouse primordial follicles *via* targeting Pyruvate Dehydrogenase Kinase Isoenzyme 1 (PDK1) and Caspase-9 [14]**.** MiRNAs and autophagy are both known to play roles in regulating endothelial functional, but the role of miR-378-3p in endothelial autophagy and function has not yet been investigated. The goal of the present study is to investigate the role of miR-378-3P in endothelial autophagy and function using a loss- and gain-of function approach. This study demonstrates for the first time that there is an inverse relationship between miR-378-3p expression and endothelial autophagy. Gain- and loss-of miR-378-3p was associated with reduced and increased endothelial autophagy and function, respectively. These findings warrant future investigation and indicate that miR-378-3p may provide a therapeutic target to modulate endothelial autophagy and function to treat CVDs.

## 2 Methods

### 2.1 Cell culture, miR-378-3p-mimic and -antagomir expression

Human umbilical vein endothelial cells [HUVECs (passage 5–7), Lonza], a common *in vitro* standard model to study ECs [15], [16], [17] were cultured in endothelial growth medium-2 (EGM™-2 Lonza™) supplemented with growth factors, serum and antibiotics at 37^o^ C in humidified 5 % CO_2_ incubator. HUVECs were transfected with mimic and antagomir to overexpress and inhibit expression of miR-378-3P, respectively, using a standard reverse transfection protocol (Lipofectamine ® 3000). To inhibit expression of miR-378-3P, a solution of 5 nm antagomir (Dharmacon™: Cat #200121) or 5 nm scrambled control (Dharmacon™: Cat # 2575450), lipofectamine (Invitrogen), and reduced serum media (Opti-MEM) were added to cells in each well. To overexpress miR-378-3P, a solution of 5 nm hsa-miR-378a-3p (human) miRIDIAN microRNA mimic (Horizon: C-300686-07-0005) or miRIDIAN microRNA mimic negative control #1 (Horizon: CN-001000-01-05), lipofectamine (Invitrogen), and reduced serum media (Opti-MEM) were added to cells in each well. RNAs or proteins were collected 24, 48 or 72-h post-transfection. For ATG7 silencing, a solution of 5 nm ATG7 silencing RNA (siATG7) (Ambion: Cat # 4392420 - s20651) or a 5 nm scrambled control (Scr) (Ambion: Cat # 4390843), lipofectamine (Invitrogen), and reduced serum media (OptiMEM) was added to cells in each well, and RNA and proteins were extracted 24-h post-transfection [18].

### 2.2 Quantitative real-time PCR

Total RNA was extracted in Trizol reagent (Invitrogen) and further separated into mRNA and miRNA using the RNeasy Mini kit (Qiagen) and RNeasy Cleanup Kit (Qiagen), respectively. RNA concentration was measured using the Nanodrop. Complementary DNA (cDNA) from mRNA was synthesized using the QuantiTech Reverse Transcription Kit (Qiagen) and cDNA from miRNA was synthesized using qScript microRNA cDNA synthesis kit (Quantabio), both followed by a polymerase chain reaction using Thermal Cycler Real-Time PCR machine (Eppendorf). Quantitative real time PCR (qPCR) for miRNA was conducted with the mixture of SYBR Select Master Mix (Applied Biosystems), forward primers of *hsa-miR-378a-3p* (Forward -5′-ACTGGACTTGGAGTCAGAAGG-3′) and *RNU6* (Forward-5′-GCAAATTCGTGAAGCGTTCC-3′), universal reverse primer (Quantabio), and cDNA as instructed by qScript microRNA cDNA Synthesis Kit. qPCR for mRNA was conducted with the mixture of SYBR Select Master Mix (Applied Biosystems), forward and reverse primers of ATG7 [18], *Protein Disulfide Isomerase Family A Member 4* (PDIA4), *Cyclin-dependent Kinase Inhibitor 1a* (p21) [15], *Vascular Endothelial Growth Factor A* (VEGFa) [19]*, Pyruvate Dehydrogenase Kinase, Isoenzyme 1* (PDK-1), *Caspase-9* (Cas-9), and GAPDH [20] (Table 1) and QuantStudio®3 Real-Time PCR Instrument (Applied Biosystems). Comparative Delta Delta CT method was employed for data analysis [21].

**Table 1:** List of primers used to amplify respective genes.

Targets	Forward primer's sequence	Reverse primer's sequence
PDIA-4	5′-CACGCTTGTGTTGACCAAAGA-3′	5′-AATTGGAGGAGAACGCTTGCT-3′
PDK1	5′-CTGTGATACGGATCAGAACCG-3′	5′-TCCACCAAACAATAAAGAGTGCT-3′
PLAU	5′- CTTCGTTTCTGCGAACTAACAGG-3	5′-GCACCACTGGGGTAAGGTTT-3′

### 2.3 Western Blot

EC lysates were collected and extracted in radioimmune precipitation assay (RIPA) buffer (Sigma) 24, 48, and 72-h post-transfection with either miR-378-3P antagomir (5 nm) or its scrambled control (5 nm) and miR-378-3P mimic (5 nm) or its scrambled control (5 nm). Equal amounts of protein were loaded on SDS-polyacrylamide gels and transferred to PVDF membrane (Thermo Fisher). Membranes were blocked for 1 h with 3 % BSA in 1× TBS and incubated overnight at 4^0^C with primary antibodies specifically targeting ATG7 (Cell Signalling #2631S), P21 (Cell Signalling #2947S), P62 (Cell Signalling #SQSTM1), Caspase-3 (Cell Signalling #9664S), LC3 (Cell Signalling #4600IAP) and GAPDH (Cell Signalling #5174S). Proteins were then incubated with Goat anti-Rabbit secondary antibody (Enzo ADI-SAB-300-*J*) for 2 h at room temperature, bands were visualized with ECL substrate using chemiluminescence channel and 700 channel in LiCor Fc Odyssey system. The bands were quantified using LiCor Fc Odyssey inbuilt software.

### 2.4 Proliferation assay

To analyze proliferation of HUVECs after transfection with mimic, antagomirs and respective controls, a colorimetric proliferation assay with WST-1 reagent was performed according to instructions from manufacture (Roche Applied Science, Rotkreuz, Switzerland, Cat. No. 11644807001). 1.5 ∗ 10^5^ cells/ml concentration per well were seeded in the 6-well assay plate and transfected with mimic and scrambled control or antagomir and scrambled control as described before. Cells were incubated with warm EGM2 in 37 °C, 5 % CO_2_ for 24 h or 48 h and collected with trypsin. 100 μl cells were reseeded in triplicates for each biological replicate into 96 well plate, and 10 μl of WTS-1 reagent was added to each well. Cells were incubated with warm EGM2 in 37 °C, 5 % CO_2_ for 4 h and absorbance was measured at 440 nm.

### 2.5 Migration assay

To analyze migratory abilities of HUVECs after transfection with mimic, antagomirs and respective controls, a scratch or migration assay was performed [22]. 1.2–1.5 ∗ 10^5^ cells/ml concentration per well were seeded in the 6-well assay plate and transfected with mimic and scrambled control or antagomir and scrambled control as described before. Cells were incubated with warm EGM2 in 37 °C, 5 % CO_2_ for 24 h or until 90 % confluency was reached. A straight scratch was made in the middle of the cell monolayer using p1000 pipette tip. Cells were imaged immediately at T_0_ using phase contrast microscopy. Cells were then put back to the incubator and imaged every 4 h up to 20 h. The percent of open wound area at each time point was calculated using Image J wound healing tool [23].

### 2.6 Tube formation assay

To evaluate the ability of ECs to adhere, migrate, differentiate, and grow, angiogenesis or tube formation assay was performed. HUVECs were transfected with mimic, antagomir or respective scrambled control as described before. *In Vitro* Angiogenesis Assay Kit (Millipore #ECM625) was used as instructed by the manufacturer [15]. Briefly, cells were cultured on the 96-well plate coated with ECMatrix provided by the kit and subsequently evaluated for tube formation abilities as instructed by the kit.

### 2.7 Statistical analysis

Data are expressed as the mean ± SD. Student's *t*-*test* was applied when the means of two groups were compared using Excel. An ANOVA with Tukey's post-hoc correction was applied when the means of more than two groups were compared using GraphPad-Prism software. A *p*-value <0.05 was considered to indicate statistical significance.

## 3 Results

### 3.1 An inverse relationship between endothelial autophagy and miR-378-3p expression

**Fig. 1:**
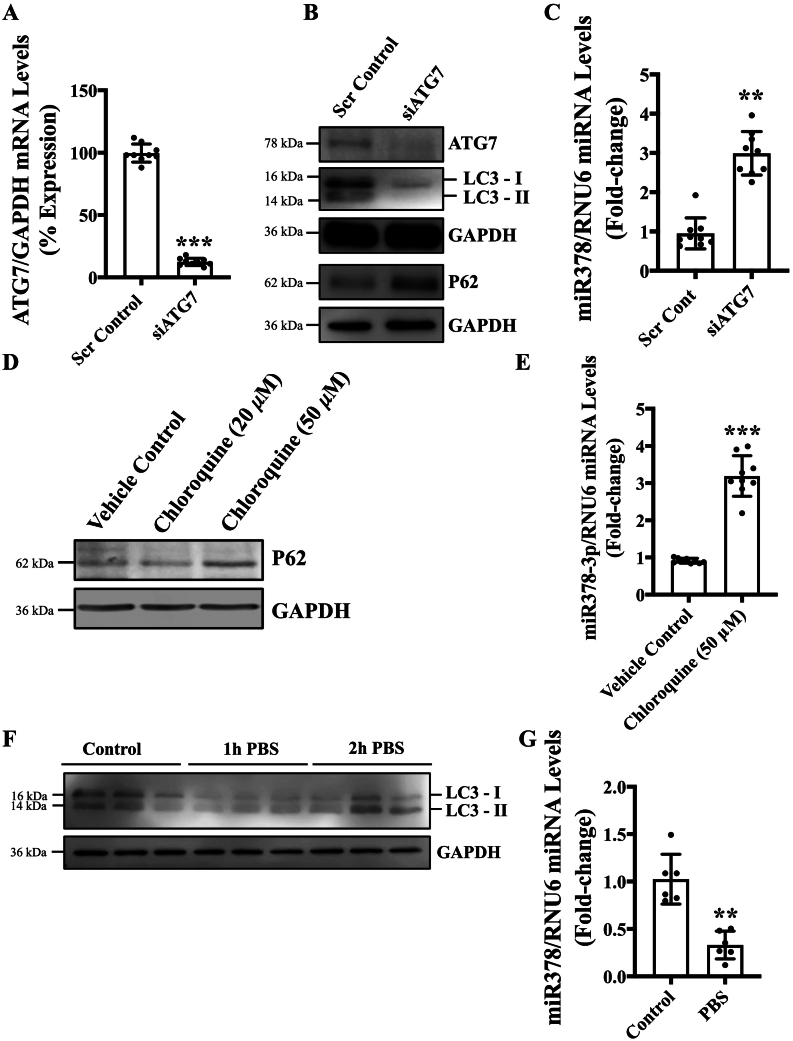
An inverse relationship between endothelial autophagy and miR-378-3p expression. **(A)** HUVECs were transfected with siATG7 for 24 h, qPCR was performed targeting ATG7, **(B)** western blot was performed targeting ATG7, LC3, P62. GAPDH and **(C)** miRNAs were extracted and expression of miR-378a-3p was measured *via* qPCR. **(D)** HUVECs were treated with chloroquine with a concentration of 20 μM or 50 μM for 24 h. Western blot targeting LC3 and P62 was performed, and **(E)** miRNA was extracted to measure the expression of miR-378a-3p *via* qPCR. **(F)** HUVECs were treated with PBS for 1 or 2-h to cause starvation-induced autophagy. Western blot was performed targeting LC3, and **(G)** miRNAs were extracted and expression of miR-378a-3p was measured *via* qPCR. Differential transcript data are presented as fold-regulation to the vehicle-treated control. Data were analyzed using Student's *t*-test and, data is presented from n (biological replicats) =3 in triplicate for qPCR except fig. G where *n* = 3 in duplicates, and *n* = 4 for western blots. Data are expressed as mean ± SD. *, **, *** represent *p* < 0.05, 0.01 or 0.001, respectively, *versus* corresponding vehicle control or scrambled control (Scr Cont).

MiRNA-378-3p has previously been shown to be associated with autophagy [14]. We further investigated the relationship between miRNA-378-3p and autophagy in ECs. To this aim, we first genetically inhibited autophagy by silencing ATG7 [18]. We confirmed the successful silencing of ATG7 at the transcript and protein level (Fig. 1**A**, **B**) in ECs, which led to inhibition of autophagy that is evident by reduced LC3-II and increased P62 accumulation in ATG7-silenced in comparison to control ECs (Fig. 1**B**). We then measured the expression level of miR-378-3p and observed significantly increased expression of miR-378-3p in ATG7-silenced in comparison to control ECs (Fig. 1**C**). To confirm that increased miR-378-3p expression in ATG7-silenced ECs is not specific to ATG7 but is due to inhibition of autophagy, we next inhibited autophagy pharmacologically using chloroquine [24] and measured autophagy as well as miR-378-3p expression. Chloroquine successfully inhibited autophagy, which is evident by increased P62 accumulation (Fig. 1**D**). Chloroquine-mediated autophagy inhibition was associated with increased miR-378-3p expression in ECs (Fig. 1**E**). Starvation activates autophagy in ECs [25]. Next, we investigated the effect of autophagy activation on miR-378-3p expression in ECs, and treated ECs with either phosphate buffered saline (PBS) or complete media, and measured autophagy and miR-378-3p expression in ECs. PBS-treatment successfully activated autophagy in ECs, as shown by increased LC3-II expression in PBS-treated in comparison to complete media-treated ECs (Fig. 1**F**). Interestingly, starvation-mediated autophagy activation led to reduced miR-378-3p expression in PBS-treated in comparison to complete medium-treated ECs (Fig. 1**G**). Overall, these results suggest an inverse relationship between autophagy activation and miRNA-378-3p expression in ECs.

### 3.2 Cause and effect relationship between miR-378-3p expression and autophagy in ECs

**Fig. 2:**
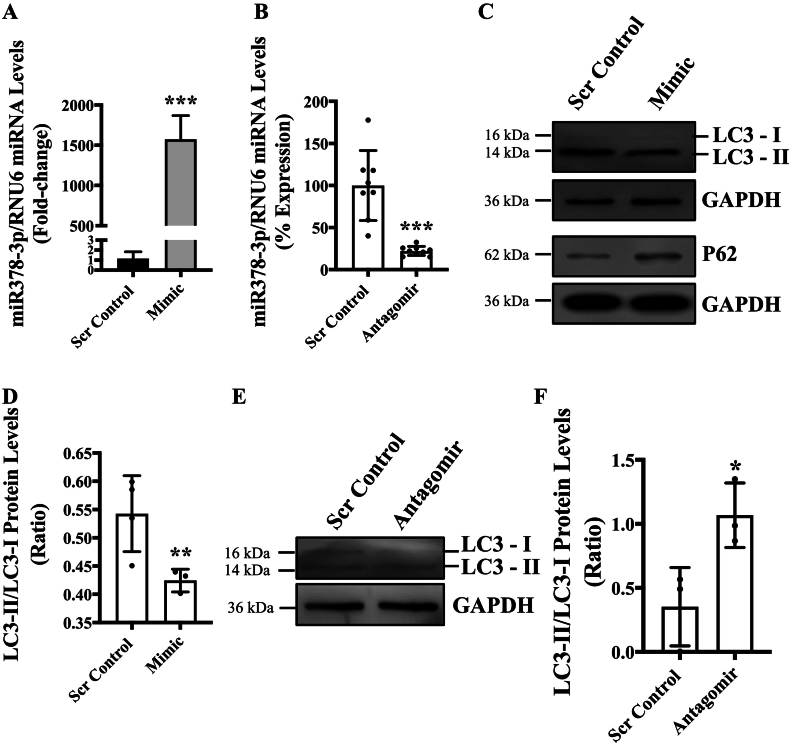
Cause and effect relationship between miR-378-3p expression and autophagy in ECs. HUVECs were reverse transfected with either mimic (5 nm), antagomir (5 nm) and respective scrambled control (5 nm) and incubated in complete EGM-2 medium for 48 h. **(A, B)** show Successful mimic-mediated miR-378-3p overexpression and antagomir-mediated miR-378-3p inhibition. **(C, D)** Proteins were extracted from HUVECs transfected with mimic, and western blot was performed targeting LC3, P62 and GAPDH, and ratio of LC3II/LC3I was quantified. **(E, F).** Proteins were extracted from HUVECs transfected with antagomir (5 nm), and western blot was performed targeting LC3 and GAPDH. Ratio of LC3II/LC3I was quantified. Data were analyzed using Student's *t*-test and, data is presented from *n* = 3 in triplicate for qPCR and n = 3–4 for western blots. Data are expressed as mean ± SD. *, **, *** represent *p* < 0.05, 0.01 or 0.001, respectively, *versus* corresponding Scr Cont.

To delineate the cause and effect relationship between miR-378-3p expression and autophagy in ECs, we overexpressed and inhibited the expression of miR-378-3p expression using mimic and antagomir, respectively, and measured autophagy in ECs. We first demonstrated the successful overexpression and inhibition of miR-378-3p in ECs using mimic and antagomir, respectively (Fig. 2**A**, **B**). Increased mimic-mediated expression of miR-378-3p was associated with significantly lower LC-II/LC3-I ratio leading to higher p62 accumulation in comparison to control ECs indicating reduced or impaired autophagy (Fig. 2**C**&**D**). Similarly, successful antagomir-mediated inhibition of miR-378-3p led to significantly higher LC3-II/LC3-I ratio in antagomir-transfected ECs in comparison to control ECs indicating autophagic activation in antagomir-transfected ECs (Fig. 2**E**, **F**). These data indicate that miR-378-3p expression level is the cause behind autophagic effect in ECs.

**Fig. 3:**
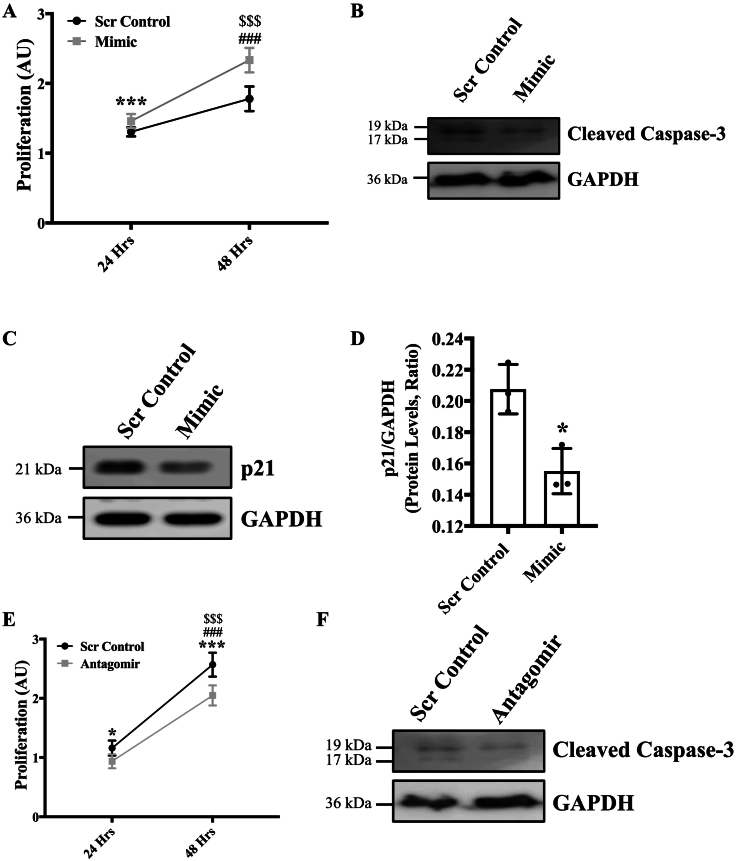
Over-expression of miR-378-3p induced endothelial cell proliferation. HUVECs were reverse transfected with either scrambled control (5 nm) or miR-378-3P mimic (5 nm). **(A)** Cells were incubated in complete EGM-2 medium for 24 h, 48 h and collected with trypsin. 100 μl of cell suspension containing equal number of cells, were reseeded in triplicates for each biological replicate into 96 well plate, and 10 μl of WST-1 reagent was added to each well. Cells were incubated with warm EGM2 in 37 °C, 5 % CO_2_ for 4 h and absorbance was measured at 440 nm. **(B)** HUVECs were transfected with mimic for 48 h and then western blot targeting cleaved caspase-3 and GAPDH was performed. HUVECs were transfected with mimic for 48 h and **(C)** qPCR and **(D)** western blot targeting p21 and GAPDH. **(E)** Cells were incubated in complete EGM-2 medium for 24 h and 48 h and collected with trypsin. 100 μl of cell suspension containing equal number of cells were reseeded in triplicates for each biological replicate into 96 well plate, and 10 μl of WST-1 reagent was added to each well. Cells were incubated with warm EGM2 in 37 °C, 5 % CO_2_ for 4 h and absorbance was measured at 440 nm. **(F)** HUVECs were transfected with antagomir for 48 h and then western blot targeting cleaved caspase-3 and GAPDH was performed. Data for fig. A and E were analyzed using 2-way ANOVA with Tukey's multiple comparison test (*n* = 3 in triplicate), and data for western blots were analyzed using student's *t*-test (n = 3). Data are expressed as mean ± SD. *, *** represent *p* < 0.05 and 0.001, respectively, *versus* Scr Cont for 24 h, ###*p* < 0.0001 *versus* mimic for 24 h, $$$p < 0.0001 *versus* Scr Cont for 48 h in fig. A and E. *p < 0.05 *versus* Scr Cont for fig. D.

**Fig. 4:**
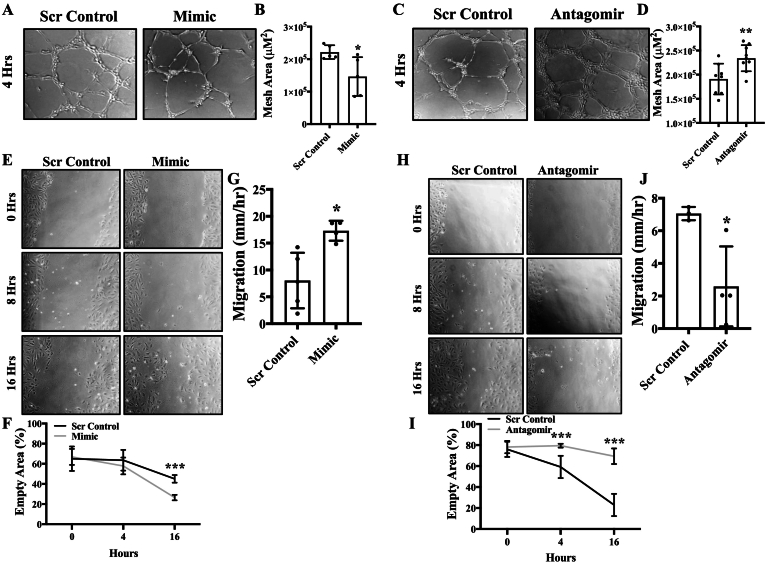
Over-expression and inhibition of miR-378-3p inhibits and promotes endothelial function, respectively. HUVECs were reverse transfected with either miR-378-3P mimic (5 nm) or antagomir (5 nm) and their respective scrambled controls (5 nm). Cells were incubated in complete EGM-2 medium for 48 h. **(A-D)***In vitro* tube formation assay was conducted by seeding cells on Matrigel to assess their ability to form tubes. Mesh area quantified using Image J angiogenesis tool (*n* = 3 in duplicates for fig. B and *n* = 4 in duplicate for fig. D). **(E-J)** A scratch was made using p200 in the middle of the cell monolayer, and cells were incubated in low serum media (MCDB131 + 1 % FBS). Cells were imaged immediately at T_0_ using phase contrast microscopy then put back to the incubator and imaged every 4 h up to 20 h. The percent of open wound area at each time point was calculated using Image J wound healing tool. Migration (the velocity of the cells to close the entire wound area) was calculated (n = 4 in triplicates and every triplicate data was averaged in the analysis). Empty area were calculated using 2-way ANOVA with Tukey's multiple comparison test (n = 4 in triplicate) in fig. F and I, all other data were analyzed using Student's *t*-test. Data are expressed as mean ± SD. *, ** represent *p* < 0.05 and < 0.01, respectively *versus* Scr Cont. In fig. E and F, *** represent *p* < 0.001*versus* Scr Cont at respective timings.

**Fig. 5:**
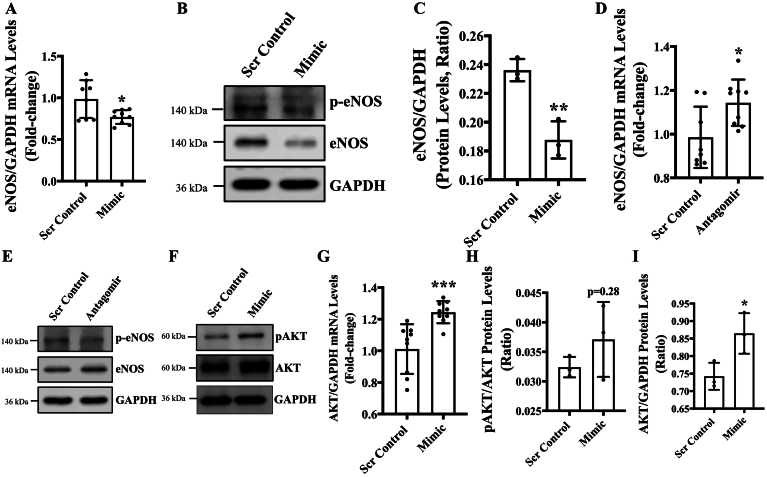
MiR-378-3p regulates endothelial function *via* modulating eNOS. HUVECs were reverse transfected with either miR-378-3P mimic (5 nm) or antagomir (5 nm) and their respective scrambled control (5 nm). Cells were incubated in complete EGM-2 medium for 48 h. **(A)** and **(D)** qPCR was performed targeting eNOS (*n* = 3 in triplicates). **(B, C, E)** Western blot targeting eNOS, p-eNOS, GAPDH. Band intensities were quantified. **(F, G, H, I)** Western blot targeting AKT, p-AKT, GAPDH was performed in the mimic group and quantified (n = 3). **(G)** qPCR targeting AKT performed in the mimic group (n = 3 in triplicates). Differential transcript (quantitative PCR) data are presented as fold-regulation to the scrambled control-treated cells. Data were analyzed using Student's t-test and data are expressed as mean ± SD. *, **, *** represent p < 0.05, 0.01 and 0.001 *versus* Scr Cont.

**Fig. 6:**
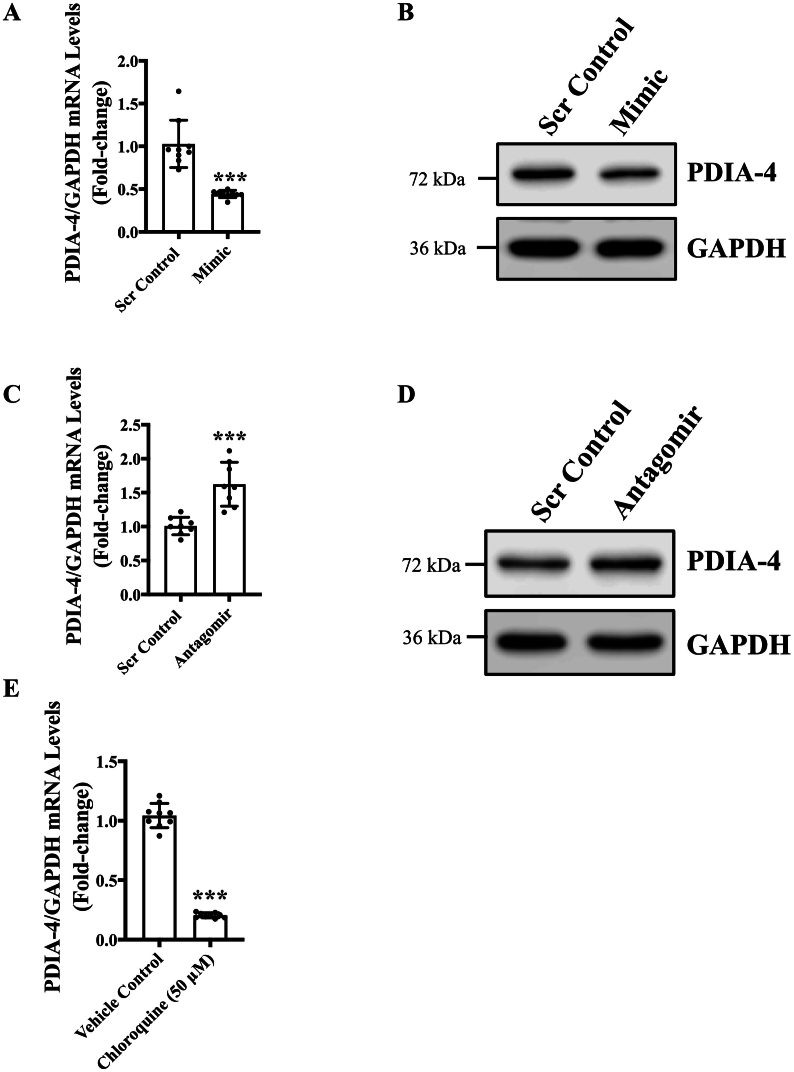
PDIA-4 is a novel target of miR-378-3p in ECs. HUVECs were reverse transfected with either miR-378-3P mimic (5 nm) or miR-378-3P antagomir (5 nm) and their respective controls and incubated in complete EGM-2 medium for 48 h. **(A-D)** qPCR (n = 3 in triplicate) and western blot (n = 3) targeting PDIA-4 and GAPDH. **(E)** HUVECs were treated with 50uM chloroquine, qPCR and western blot were performed targeting PDIA-4 and GAPDH (n = 3 in triplicate for qPCR and n = 3 for western blot). Differential transcript (quantitative PCR) data are presented as fold-regulation to the scrambled control-treated cells. Data were analyzed using Student's t-test and data are expressed as mean ± SD. *** represents p < 0.001 *versus* respective vehicle or Scr Cont.

**Table 2:** Identification of miR378-3p-target genes in endothelial cells using TargetScan.

Ranking	TargetScan	Gene	Score
1	PDIA4	Protein Disulfide Isomerase Family A Member 4; Erp70; Erp72	−0.63
2	IMPG1	Interphotoreceptor Matrix Proteoglycan 1	−0.63
3	TMED7-TICAM2	TMED7-TICAM2	−0.61
4	SDAD1	SDA1 Domain Containing 1	−0.6
5	GOLT1A	Golgi Transport 1A	−0.57
6	FAM179B	TOG Array Regulator of Axonemal Microtubules 1	−0.54
7	PAG1	Phosphoprotein Associated with Glycosphingolipid-Enriched Microdomains 1	−0.53
8	RSPH4A	Radial Spoke Head Component 4A	−0.53
9	KIAA1429	KIAA1429	−0.5
10	CD226	CD226 Antigen	−0.48

### 3.3 Over-expression and inhibition of miR-378-3p inhibits and promotes endothelial function, respectively

Next, we investigated the effect of overexpression or inhibition of miR-378-3p on endothelial function by measuring main indices of endothelial function such as, cell proliferation, migration and tube-forming potential of ECs [15] [16] [17]. To this aim, HUVECs were transfected with either mimic or scrambled-control, and cell proliferation was assessed using a colorimetric proliferation reagent WST-1. Cell proliferation was significantly increased in mimic-transfected ECs in comparison to control ECs (Fig. 3**A**). To confirm that reduced cell proliferation is not associated with reduced viability or increased apoptosis, we measured viability using Cytosmart cell counter and apoptosis *via* immunoblotting for cleaved caspase-3 and by flow cytometry. Viability was not affected (Supp. Fig. 1A; supplemental material is available at https://doi.org/10.6084/m9.figshare.20361717.v1) and cleaved-caspase 3 expression was also not affected indicating apoptosis was not affected in mimic-transfected ECs (Fig. 3**B**). These results show that increased cell proliferation in mimic-transfected ECs was not associated with apoptosis. We then measured the expression level of the cell cycle inhibitor p21. P21 is a cyclin-dependent kinase inhibitor that is responsible to promote cell cycle arrest and it inhibits endothelial cell proliferation [26]. Our qPCR and immunoblotting data for p21 showed significantly decreased expression in mimic-transfected ECs in comparison to control ECs (Fig. 3**C**, **D**) indicating increased cell proliferation in mimic-transfected ECs. In contrast, cell proliferation was significantly reduced in ECs transfected with antagomir compared to scrambled controls (Fig. 3**E**). Cleaved caspase-3 expression did not alter in antagomir transfected ECs indicating apoptosis was not affected (Fig. 3**F**). We then evaluated the ability of ECs to form tube-like structures representing angiogenesis *in vitro* on Matrigel. ECs transfected with mimic formed fewer and weaker tubes with significantly less mesh area compared to control cells (Fig. 4**A**, **B**). Contrastingly, ECs transfected with antagomir formed more tubes with significantly more mesh area compared to control ECs (Fig. 4**C**, **D**), indicating that miR-378-3p expression inversely effects tube-forming potential of ECs. To assess the ability of cells to repair wounds, a scratch assay was conducted. ECs transfected with mimic showed a significantly smaller percentage of open wound area and significantly higher migration rate compared to control indicating enhanced migratory potential (Fig. 4**E**-**G**). An opposing trend was observed for cells transfected with antagomir, which had a significantly larger percentage of open wound area and significantly lower migration rate compared to controls indicating impaired migration (Fig. 4**H**-**I**), and direct correlation between miR-378-3p expression and migratory potential of ECs.

### 3.4 MiRNA-378-3p regulates endothelial function is mediated by eNOS

To evaluate the mechanisms behind miR-378-3p expression and observed endothelial phenotype, we measured the transcript and protein level of endothelial Nitric Oxide Synthase (eNOS). eNOS mediated nitric oxide production that directly regulates endothelial function [27]. Our data showed significantly reduced eNOS expression and activation in mimic-transfected ECs in comparison to control ECs (Fig. 5**A**-**C**). As expected, eNOS was found to be upregulated both on the transcript and protein level in antagomir-transfected ECs compared to control (Fig. 5**D**, **E**). The serine/threonine kinase AKT activates eNOS leading to increased NO production [28]. Interestingly, AKT was significantly upregulated in mimic-transfected cells, however, the p-AKT/AKT ratio was not affected (Fig. 5**G**, **H**). AKT up-regulation was confirmed at the transcript level in mimic-transfected ECs confirming that increased transcription was the reason behind increased AKT protein expression in mimic-transfected ECs (Fig. 5**I**). Vascular endothelial growth factor a (VEGFa), which is a key regulator of angiogenesis [29], was not affected in either mimic or antagomir-transfected ECs, in comparison to control ECs (Supp. Fig. 1B).

### 3.5 PDIA-4 is a novel target of miR-378-3p in endothelium

To find the potential targets of miR-378-3p in ECs, already known targets such as VEGF-a [9], Caspase-9 [14] and PDK-1 [14] were tested *via* qPCR. None of these targets were differentially expressed in mimic- or antagomir-transfected in comparison to control ECs (Supp. Fig. 1B-E). Next, TargetScan software was used to scan for potential targets, which identified a number of proteins listed in Table 2 (Table 2). TargetScan predicts possible targets of miRNA by searching for the conserved sites that match the seed region of the miRNA [30]. PDIA-4 was identified as the top-ranking target with the lowest weighted context score −0.63, and its expression was subsequently measured in mimic- or antagomir-transfected ECs and control ECs. Our qPCR and immunoblotting data confirmed basal expression of PDIA-4 in ECs, which was significantly reduced in miR-378-3p mimic-transfected ECs and increased in antagomir-transfected HUVECs, respectively (Fig. 6**A**-**D**). In addition, chloroquine-treatment which inhibited autophagy and up-regulated miR-378-3p expression was associated with reduced PDIA-4 expression in chloroquine-treated ECs in comparison to control ECs (Fig. 6**E**). These data clearly indicate that PDIA-4 is a direct target of miR-378-3p in regulation of endothelial autophagy.

## 4 Discussion

The main observation of this study is that miR-378-3p regulates autophagy and function in ECs. MiRNAs are shown to play critical roles in autophagy and endothelial functions [3], however, this is the first molecule reported to elicit both effect in ECs. It is also important to note that the known targets of miR-378-3p such as VEGF-a [9], Caspase-9 [14] and PDK-1 [14] were not affected in ECs following modulation of miR-378-3p expression, and we identified a novel miR-378-3p target in ECs, which indicates a differential and context-dependent role played by miR-378-3p in different cell-types.

Silencing *ATG7* was chosen as a method to genetically inhibit autophagy because ATG7 is critical for autophagosome formation through two pathways [5], and loss of ATG7 has been shown to inhibit autophagy in ECs [18]. Pharmacologic inhibition of autophagy was also conducted to ensure that results observed following the silencing of *ATG7* are an effect specific to loss of autophagy instead of a downstream effect of the silencing *ATG7*. Chloroquine is a drug that has been used in multiple studies to inhibit autophagic flux and was shown to inhibit autophagy through preventing fusion of the lysosome with the autophagosome [31]. Loss of ATG7 and chloroquine, both inhibit autophagy in ECs *via* different mechanisms [18], [31], nonetheless, autophagy inhibition led to increased miR-378-3p expression, indicating that this effect is not pathway specific, but is due to inhibition of global autophagy (Fig. 1**A**-**E**). To induce autophagy, we starved HUVECs in PBS for either 1 or 2-h, which is shown to activate autophagy [5]. Starvation-induced autophagy inhibited miR-378-3p expression that again confirmed that this response is not specific to any mechanism but to activation or inhibition of global autophagy; and established an inverse relationship between miR-378-3p expression and autophagy (Fig. 1**F**, **G**).

Next, to investigate whether expression of miR-378-3p regulates autophagic activity or it is autophagy which regulates miR-378-3p expression in ECs, we overexpressed and inhibited miR-378-3p expression *via* mimic and antagomir, respectively. We observed significantly reduced and increased autophagy in mimic and antagomir-transfected ECs, respectively, which confirmed that miR-378-3p is upstream in autophagic signalling and regulates autophagic activity in ECs (Fig. 2). Overall, these findings establish a “cause and effect” relationship that miR-378-3p expression level is the cause and autophagic activity in ECs is the effect. Contrasting to our finding, Sun *et al* and Li *et al* found that miRNA-378-3p overexpression promoted autophagy and inhibited apoptosis in mouse primordial follicles and myocytes in mouse skeletal muscle, respectively [14], [32]. MiR-378-3p is highly conserved in mice and humans [9], however, its plausible that miR-378-3p elicits different responses in different cell-types by targeting different sets of genes in a context-dependent manner.

To determine the effect of miR378-3p overexpression on endothelial functions, three main indices of endothelial function, such as proliferation, migration and angiogenic potential were evaluated. We observed an inverse relationship between miR-378-3p expression and EC proliferation (Fig. 3**A**&**E**), which was independent of apoptosis (Fig. 3**B**&**F**). P21 is a cyclin-dependent kinase inhibitor that inhibits G1/S cell cycle progression and regulates cell proliferation in different cells including ECs and p21 is inversely associated with cell proliferation [26]. P21 is increasingly being linked to cell migration, cytoskeletal reorganization and cell differentiation [33]. P21 is also shown to induce autophagy in fibroblasts indicating an intricate interplay between p21, autophagy and cell cycle regulation [34]. In this study, miR-378-3p overexpression was associated with reduced p21 expression in ECs (Fig. 3**C**, **D**) which corresponds to the increased cell proliferation observed. It has been shown that when autophagic activity is impaired or decreased, there will also be a decrease in mitophagy, which is the process of degrading damaged mitochondria leading to increased reactive oxygen species production [35]. Increased oxidative stress could lead to activation of pro-apoptotic pathways and increased cell death [36]. An imbalance of cell proliferation and cell death is the cause of many pathological diseases including atherosclerosis [37], [38]. In the current study, it would be interesting to investigate how p21 participates in the autophagic process. On the other hand, cell proliferation was shown to be significantly reduced in cells downregulating miR-378-3p expression, and this decrease in cell proliferation is not associated with apoptosis (Fig. 3**F**). Overall, the result reveals an inverse relationship between miR-378-3p expression and cell proliferation in ECs.

An angiogenesis assay was performed to evaluate the ability of ECs to form tube-like structures on Matrigel. It was found that the tubes formed from mimic-transfected cells were weaker with significantly less mesh area compared to control cells (Fig. 4**A**&**B**) indicating that miR-378-3p overexpression impairs the angiogenic potential of ECs. Contrastingly and as expected, antagomir-transfected ECs formed stronger tubes and formed significantly more mesh area compared to control (Fig. 4**C**&**D**). C-Myc is a key regulator in cell transformation in tumorigenesis and it has been shown that C-Myc directly activates miR-378 which cooperates with HER2 to promote cellular transformation [39]. To evaluate the migratory ability of cells after overexpression or inhibition of miR378-3p, a scratch assay was performed. It was found that cells transfected with mimic showed a smaller percent of open area and significantly higher migration rate compared to control cells meaning increased migratory ability (Fig. 4**E**-**G**). An opposite trend was observed in antagomir-transfected ECs (Fig. **H**-**J**). Increased migration is associated with EC activation under stress conditions [40]. Although there is no information about how miR-378-3p affects migration in ECs, miR-378-3p overexpression is shown to promote migration in cancer cells. Cui *et al* reported that miR378-3p overexpression increased cell migration of oral squamous carcinoma cells and inhibition had exactly the opposite effect [41]. Chen *et al* reported that miR-378 expression increased cell migration and invasion in non-small lung cancer [42]. Ma *et al* reported that miR-378 promoted cell migration in liver cancer [43]. These reports support our finding and indicate that miR-378-3p can be a therapeutic target to not only regulate autophagy but also cell migration, which play important roles in many disease progressions, such as cancer and CVDs.

To find out potential mechanisms behind the effect of miR-378-3p on endothelial function, transcript levels of endothelial nitric oxide synthase (eNOS) was measured. We observed a significant reduction and upregulation of eNOS in mimic or antagomir transfected cells, respectively (Fig. 5**A**-**E**). Our *in-silico* findings using Target scan did not identify a binding sequence for miR-378-3p on eNOS transcript, however, interaction between promoter or intronic region of eNOS and miR-378-3p cannot be ruled out. eNOS generates the vasoprotective molecule nitric oxide to help the ECs maintain vascular function and fight vascular diseases [44]. Thus, eNOS is important in maintaining vascular homeostasis, and its downregulation is associated with endothelial dysfunction [45], which is consistent with reduced and enhanced angiogenic potential in mimic- and antagomir-transfected ECs, respectively (Fig. 4**A**-**D**). AKT as a protein kinase regulates eNOS activity by phosphorylating eNOS at Ser1177 [28], [46]. Accordingly, AKT and p-AKT were also measured, where AKT expression was increased but AKT activation appears to be un-affected in mimic-transfected ECs (Fig. 5**F**-**I**). We observed a trend (*p* = 0.28) towards an increased AKT-phosphorylation (Fig. 5**H**), which might play a role in increased EC migration observed in miR-378-3p overexpressing cells as reported by Morales-Ruiz *et al*, who showed that constitutive AKT expression in microvascular ECs could initiate and promote cell migration in the absence of VEGFa [47]. We did not observe an effect of miR-378-3p expression on VEGFa expression in ECs (Supp. Fig. 1B&C).

Finally, to find potential targets of miR-378-3p, we first evaluated the known targets from the literature. Sun *et al* recently found that miR-378-3P targeted PDK-1 to activate autophagy and inhibited apoptosis by targeting caspase-9 in primordial follicles [14]. Thus, expressions of PDK-1 and Cas-9 were measured but no significant association was observed with miR-378-3p expression level (Supp. Fig. 1D, E). Later, TargetScan software was used to identify potential targets *in silico* [30]*,* and PDIA-4 was identified as the top-most candidate (Table 2). The potential target site was also identified and the miR-378-3p binding site was observed on the 3′UTR region on PDIA-4. Our qPCR and immunoblotting data for PDIA-4, performed on mimic- and antagomir-transfected ECs showed significantly reduced and increased expression, respectively, further confirming PDIA-4 as a target for miR-378-3p in ECs (Fig. 6**A**-**D**). PDIs play essential roles in the maintenance of proteostasis and have been increasingly studied for their roles in cancer progression and metastasis [48]. In the endoplasmic reticulum, PDIs catalyze the formation of disulfide bonds to help protein fold into its final conformation [49]. PDIA-4 belongs to the PDI family and functions both as oxidoreductase and chaperone for eukaryotic proteins [50]. Accumulation of misfolded protein can lead to endoplasmic reticulum stress response (ERS response) [51], which could stimulate formation of autophagosome and triggers autophagy. Clinically, an abnormal level of PDIA-4 expression has been observed in breast, liver and thyroid cancers [52]. There is currently no information about the molecular link between PDIA-4, endothelial function and autophagy. However, Kyani *et al* reported that inhibiting PDIA-4 in brain cancer could lead to autophagy-mediated ferroptosis and could potentially become a treatment for glioma [53]. Here we found that under chloroquine inhibited autophagy, PDIA-4 was significantly downregulated, combined with the result before that autophagy inhibition led to significantly increased miR-378-3p expression, it is reasonable to conclude that there is an intricate interplay between miR-378-3p, PDIA-4 and autophagy requiring further studies.

Emerging information suggest that there is a link between microRNAs, autophagy, and disease. Several miRNAs have been shown to have key regulatory roles in a variety of autophagic processes. In prostate cancer cells, miR-26b inhibited autophagy through targeting ULK2 [54]. MiR-195 inhibition in human endothelial progenitor cells promoted autophagy through targeting GABA type A receptor associated protein like 1 [55]. Due to the vast number of diseases that arise from endothelial dysfunction, it is important to understand how miRNAs affect transcription and translation of functional genes in the endothelium to gain further insight on these diseases, and shine light on the future development of clinical treatments. This is the first study that investigates the novel role of miR-378-3p specifically in endothelial autophagy and endothelial function. The results from this study add to the current knowledge of the relationship between miRNA and endothelial function and revealed a novel target of miRNA-378-3p, warranting future investigation.

The following is the supplementary data related to this article.

Supplementary Fig. 1 (A) HUVECs were reverse transfected with either miR-378-3P mimic (5 nm) or scrambled control (5 nm). Cells were incubated in complete EGM-2 medium for 24, 48 and 72 h, and viability was calculated using Cytosmart. (B–E) HUVECs were reverse transfected with either miR-378-3P mimic (5 nm) or antagomir (5 nm) and their respective scrambled control (5 nm). Cells were incubated in complete EGM-2 medium for 48 h. qPCR was performed targeting Vegfa, PDK-1, Cas-9 and GAPDH. Differential transcript (quantitative PCR) data are presented as fold-regulation to the scrambled control-treated cells. *p < 0.05 *versus* corresponding the control. *N* = 3 in triplicate. Supplementary Fig. 1

Supplementary material Image 1

## Sources of funding

Funding for this project was provided by the Project Grant (FRN # 153216), 10.13039/501100000024Canadian Institutes of Health Research, Canada to KS. KS is also the recipient of the 2018/19 National New Investigator Award- Salary Support from the 10.13039/100004411Heart and Stroke Foundation of Canada, Canada.

## Declaration of competing interest

The authors declare that they have no known competing financial interests or personal relationships that could have appeared to influence the work reported in this paper.
