# Supplementary material for: MicroRNA miR-378-3p is a novel regulator of endothelial autophagy and function
Source: J Mol Cell Cardiol Plus. 2022 Dec 8;3:100027. doi: 10.1016/j.jmccpl.2022.100027 (PMC11708318; doi:10.1016/j.jmccpl.2022.100027)
Supplement: Supplementary Fig. 1 — (A) HUVECs were reverse transfected with either miR-378-3P mimic (5 nm) or scrambled control (5 nm). Cells were incubated in complete EGM-2 medium for 24, 48 and 72 h, and viability was calculated using Cytosmart. (B–E) HUVECs were reverse transfected with either miR-378-3P mimic (5 nm) or antagomir (5 nm) and their respective scrambled control (5 nm). Cells were incubated in complete EGM-2 medium for 48 h. qPCR was performed targeting Vegfa, PDK-1, Cas-9 and GAPDH. Differential transcript (quantitative PCR) data are presented as fold-regulation to the scrambled control-treated cells. *p < 0.05 versus corresponding the control. N = 3 in triplicate. [file mmc1.docx]

**SUPPLEMEMNTARY FIGURE 1**

**** **A B C**

**D E**

**Supplementary Figure 1.** **(A)** HUVECs were reverse transfected with either miR-378-3P mimic (5nm) or scrambled control (5nm). Cells were incubated in complete EGM-2 medium for 24, 48 and 72 hrs, and viability was calculated using Cytosmart. **(B)** and **(C)** HUVECs were reverse transfected with either miR-378-3P mimic (5nm) or antagomir (5nm) and their respective scrambled control (5nm). Cells were incubated in complete EGM-2 medium for 48 hrs. qPCR was performed targeting Vegfa, PDK-1, Cas-9 and GAPDH. Differential transcript (quantitative PCR) data are presented as fold-regulation to the scrambled control-treated cells. *p<0.05 *versus* corresponding the control. N = 3 in triplicate.
