# Supplementary material for: MicroRNA miR-378-3p is a novel regulator of endothelial autophagy and function
Source: J Mol Cell Cardiol Plus. 2022 Dec 8;3:100027. doi: 10.1016/j.jmccpl.2022.100027 (PMC11708318; doi:10.1016/j.jmccpl.2022.100027)
Supplement: Supplementary file 2 — Supplementary material [file mmc2.pdf]

Membrane 1

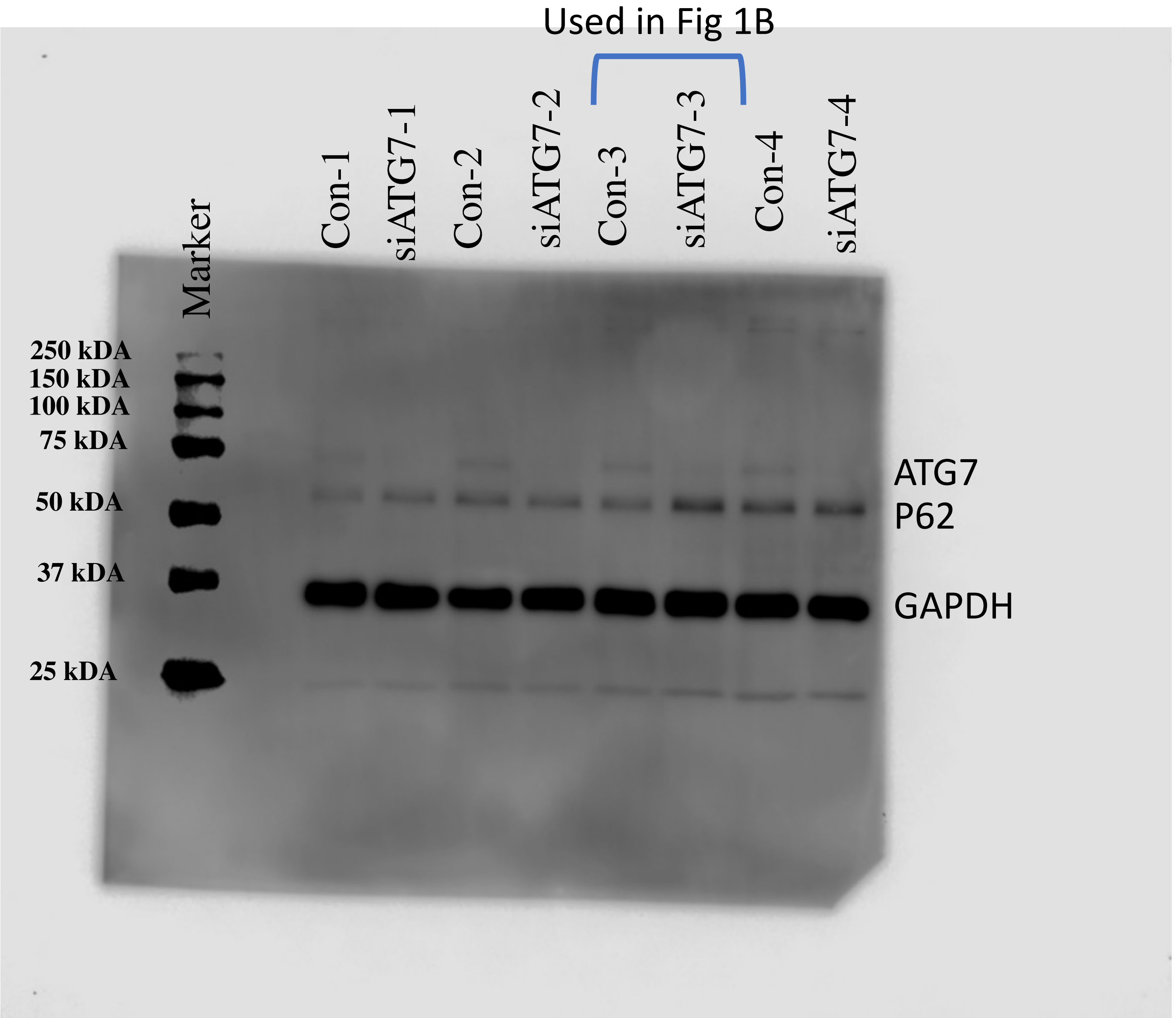


Western blot was first probed with ATG7, then P62 and then GAPDH antibody.

Bands were visualized with ECL substrate using chemiluminescence channel in LiCor Fc Odyssey system.

Membrane 2

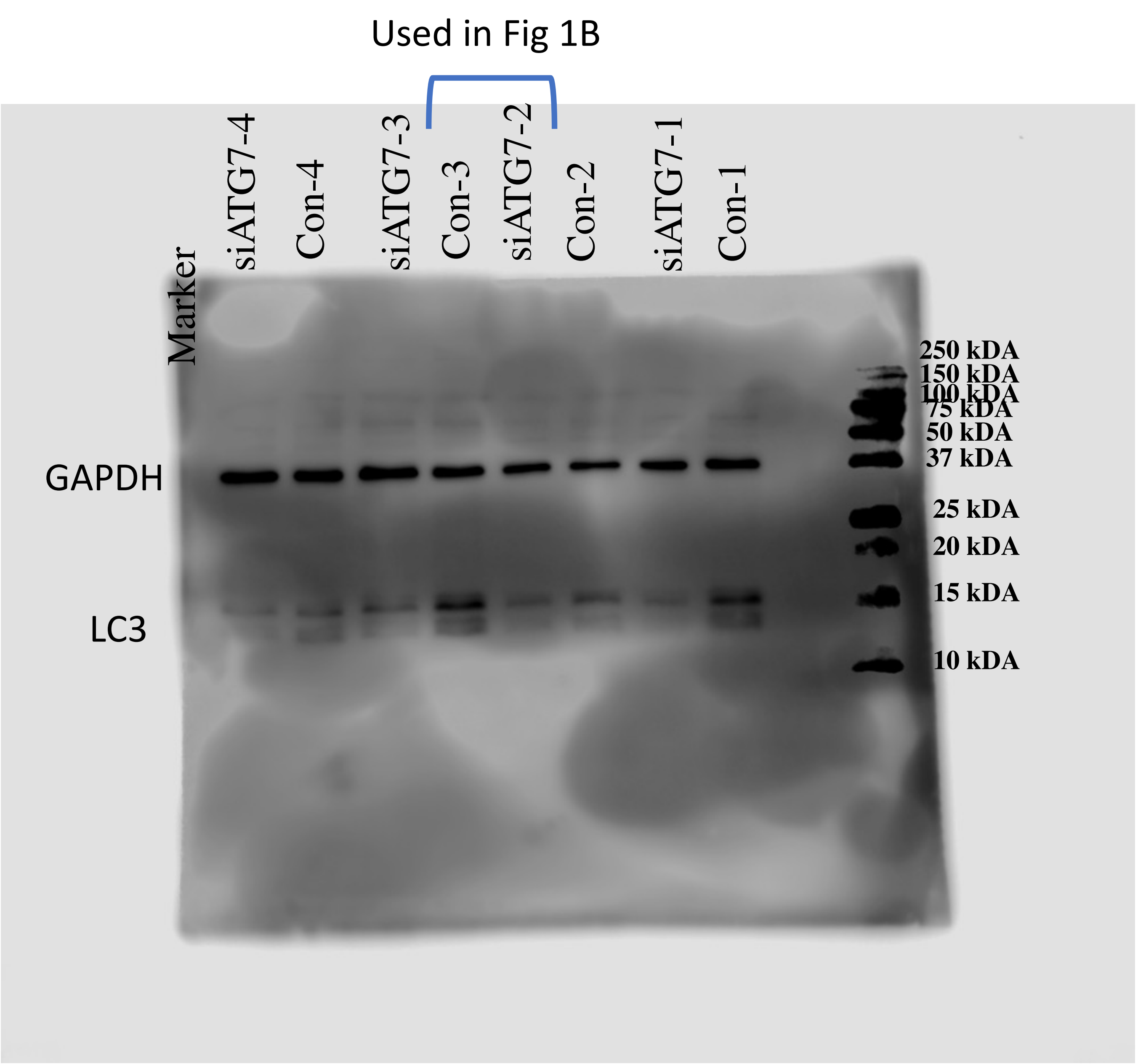


Western blot was first probed with LC3 then GAPDH antibody.

Bands were visualized with ECL substrate using chemiluminescence channel in LiCor Fc Odyssey system.

Membrane 3

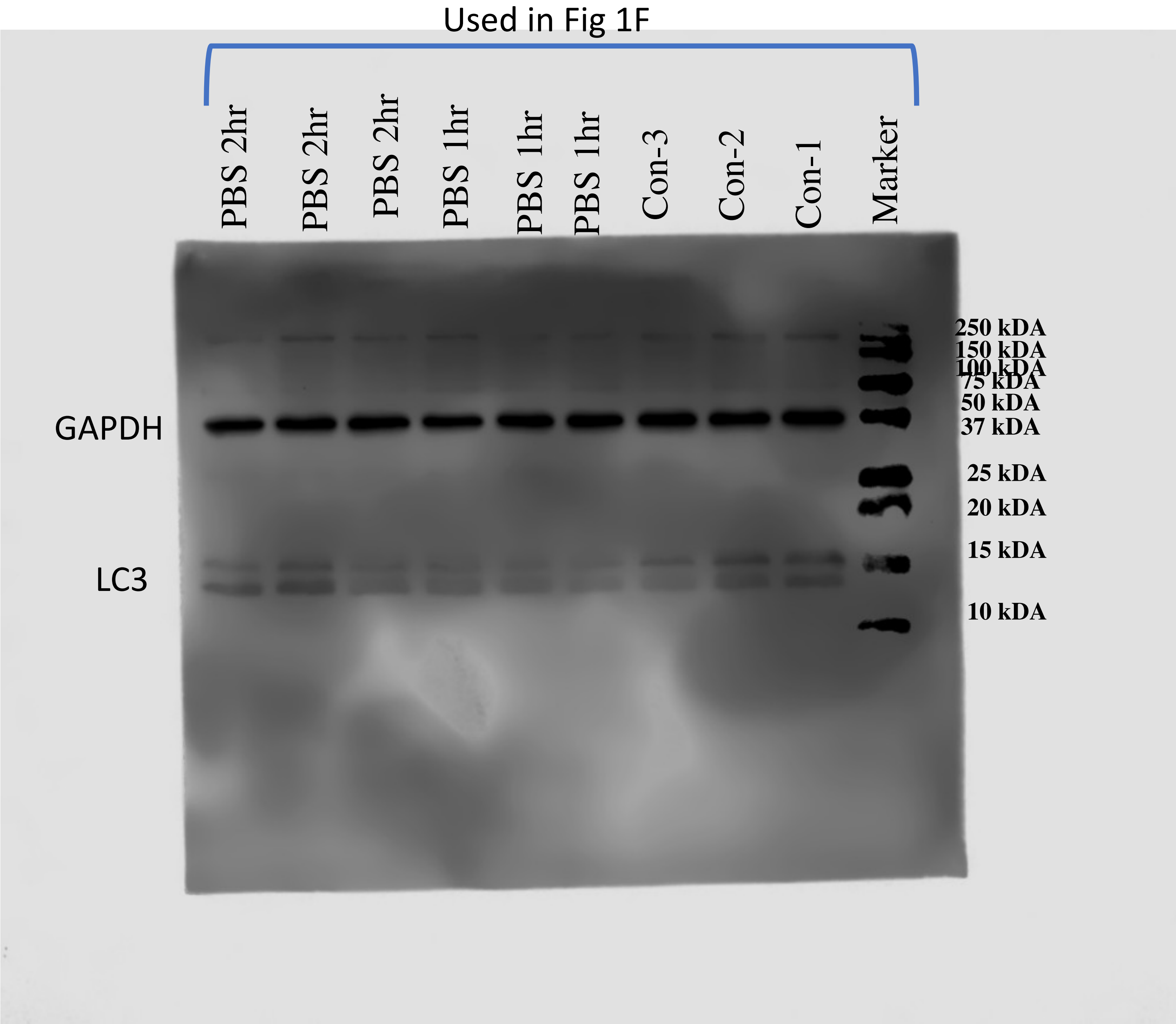


Western blot was first probed with LC3 then GAPDH antibody.

Bands were visualized with ECL substrate using chemiluminescence channel in LiCor Fc Odyssey system.

Membrane 4

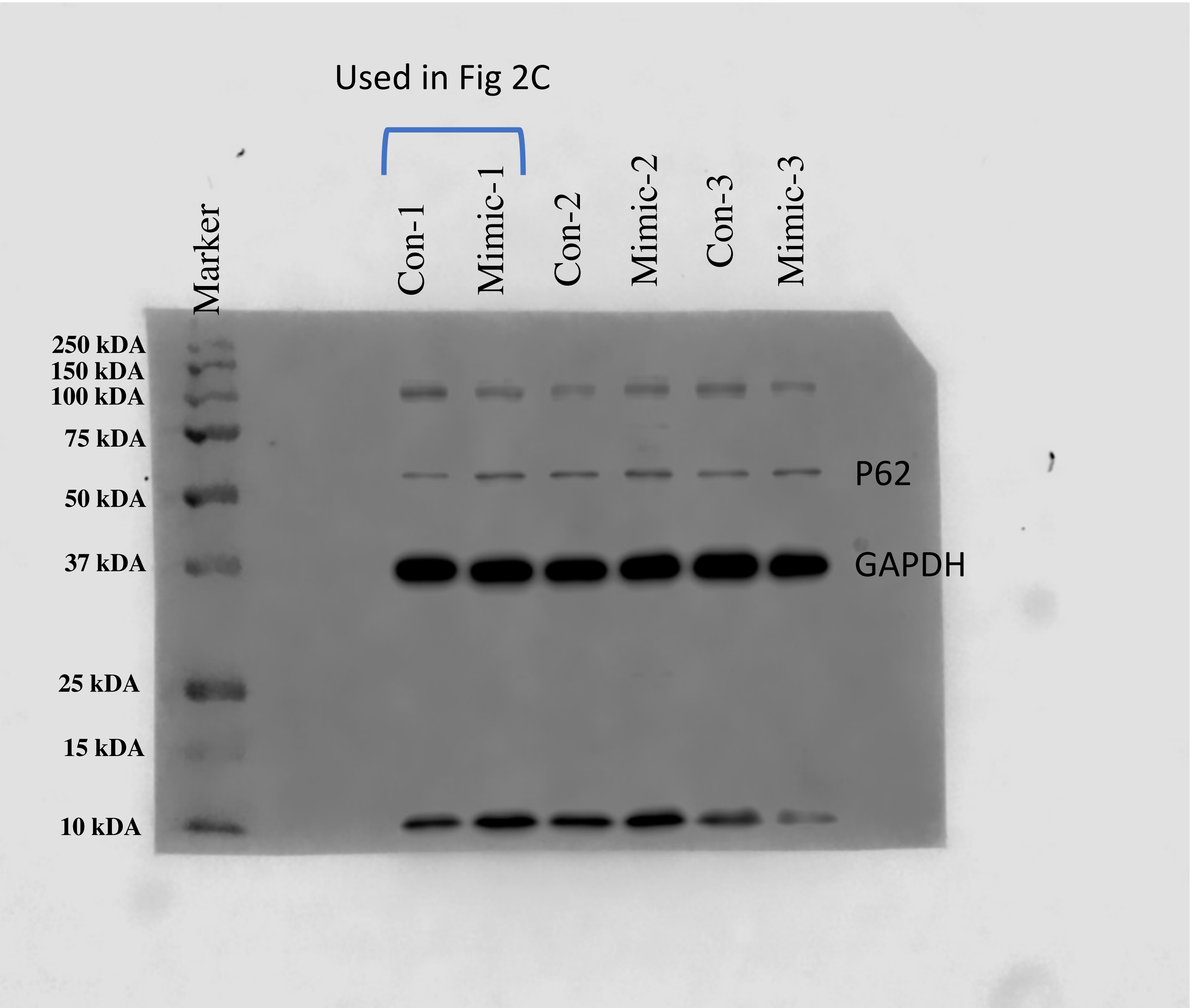


Western blot was first probed with P62 then GAPDH antibody.

Bands were visualized with ECL substrate using chemiluminescence channel in LiCor Fc Odyssey system.

Membrane 5

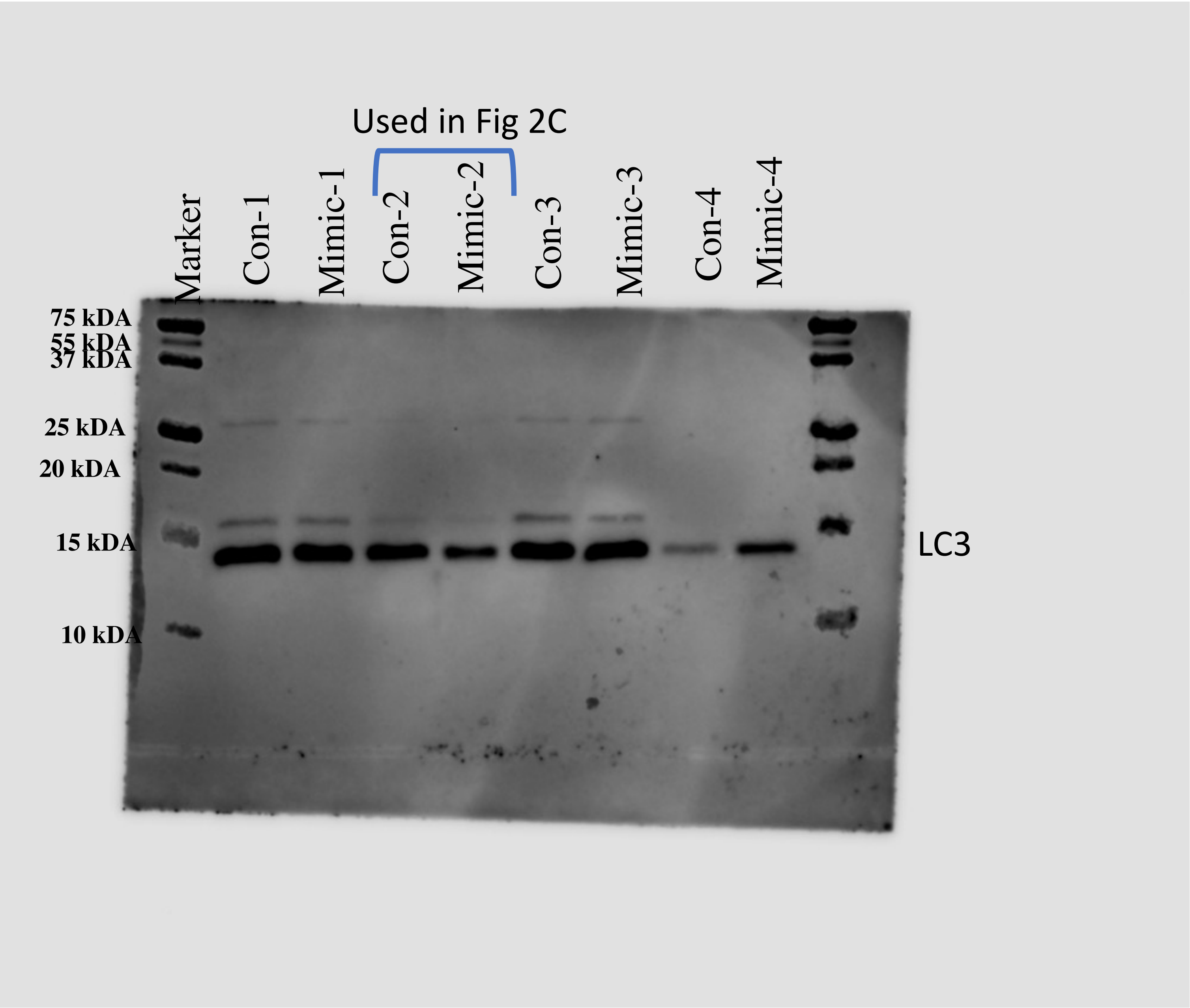


Western blot was first probed with LC3 then GAPDH antibody.

Bands were visualized with ECL substrate using chemiluminescence channel in LiCor Fc Odyssey system.

Membrane 5

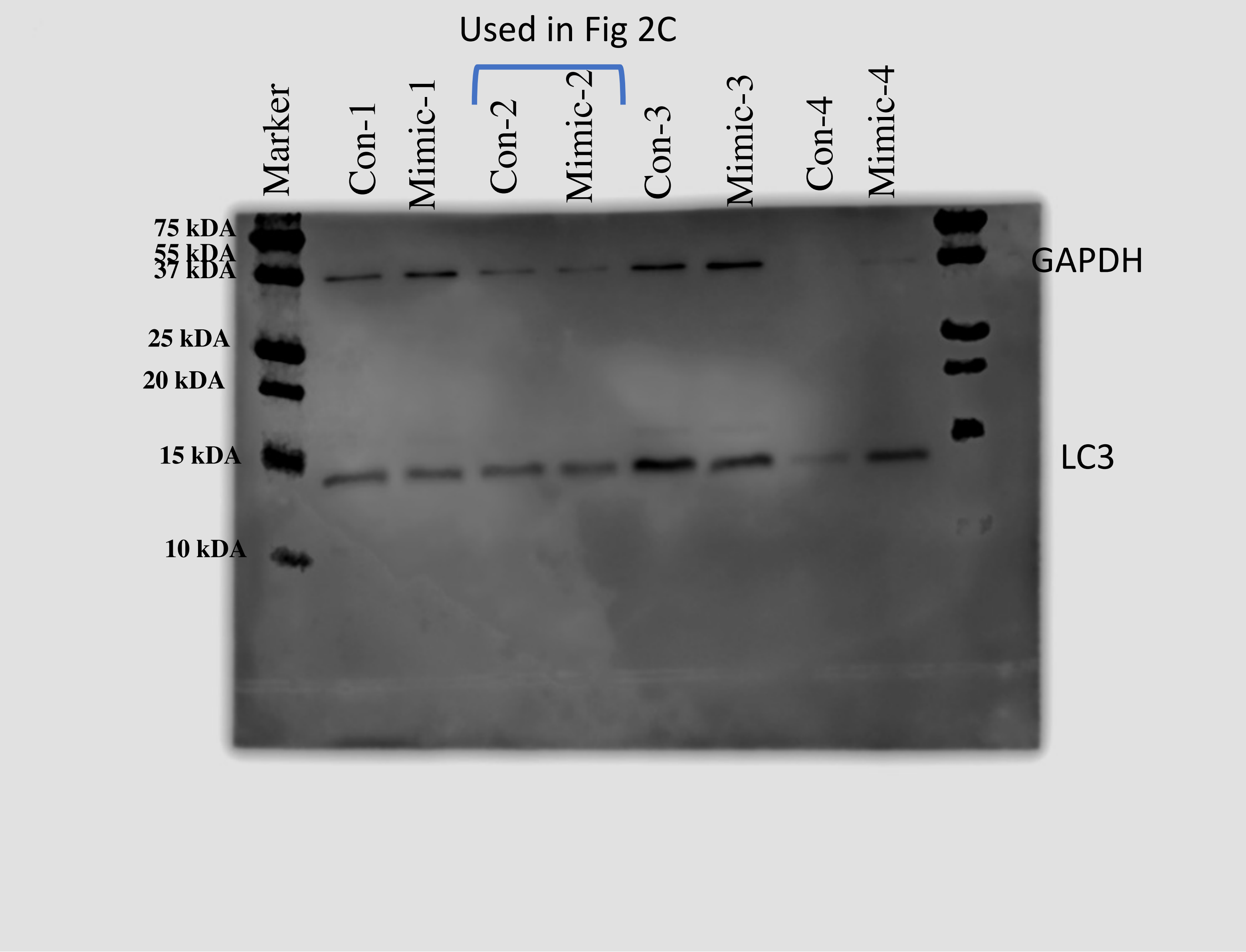


Bands were visualized with ECL substrate using chemiluminescence channel in LiCor Fc Odyssey system.

Membrane 6

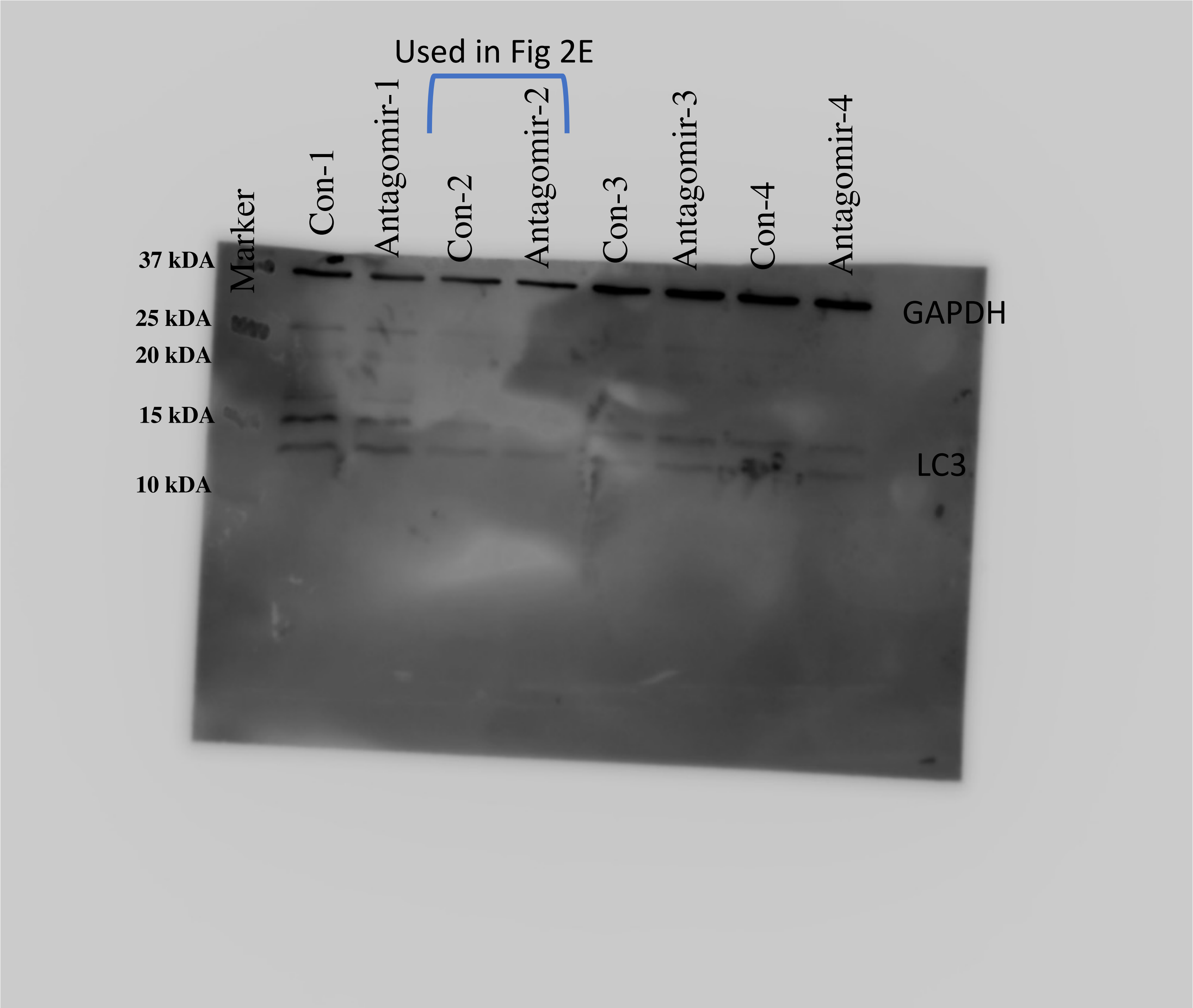


Western blot was first probed with LC3 then GAPDH antibody.

Bands were visualized with ECL substrate using chemiluminescence channel in LiCor Fc Odyssey system.

Membrane 7

Membrane 7 was cut below 37kDA in order to incubate cleaved caspase 3 antibody and GAPDH on the same day

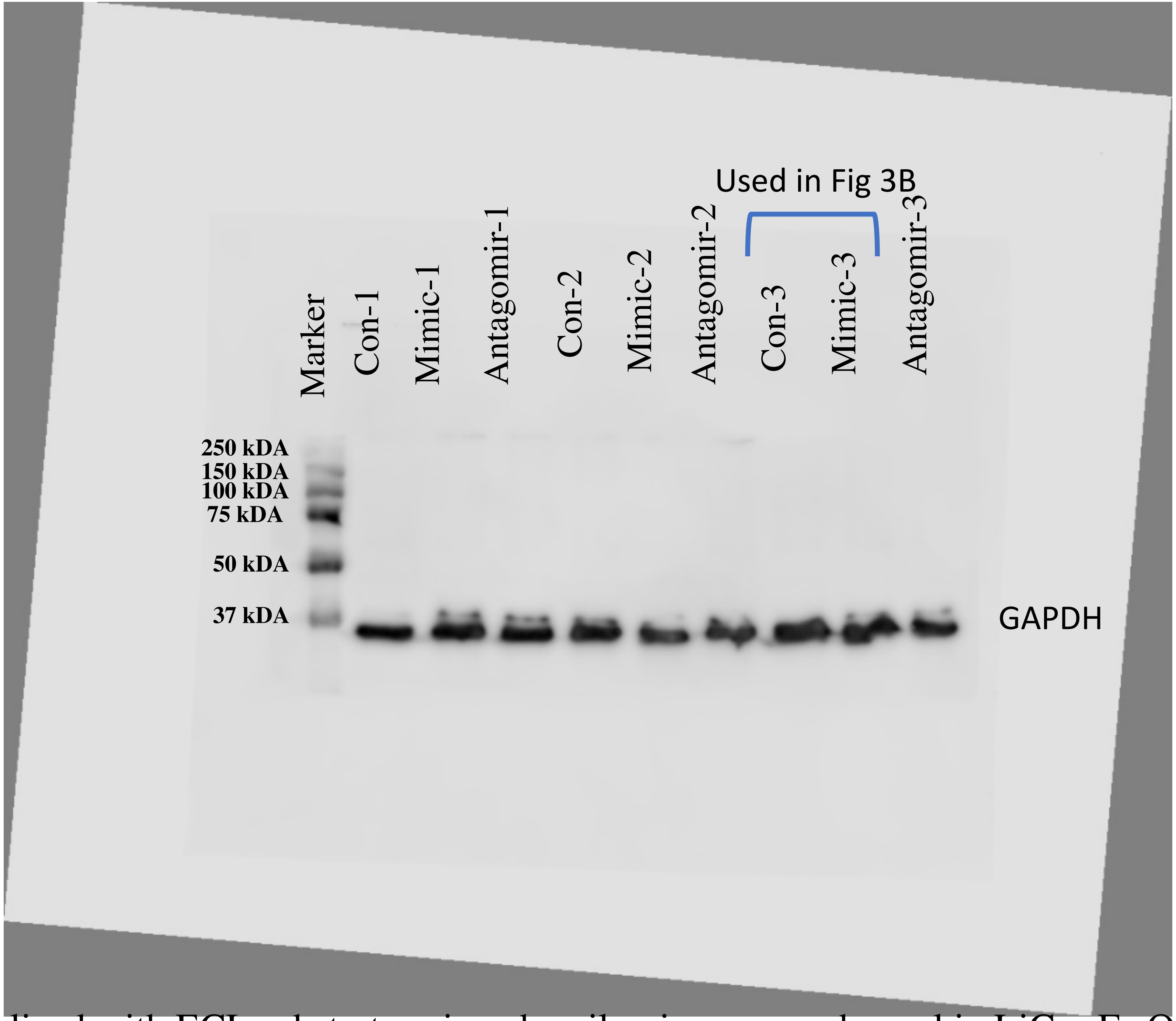


Bands were visualized with ECL substrate using chemiluminescence channel in LiCor Fc Odyssey system.

Membrane 7

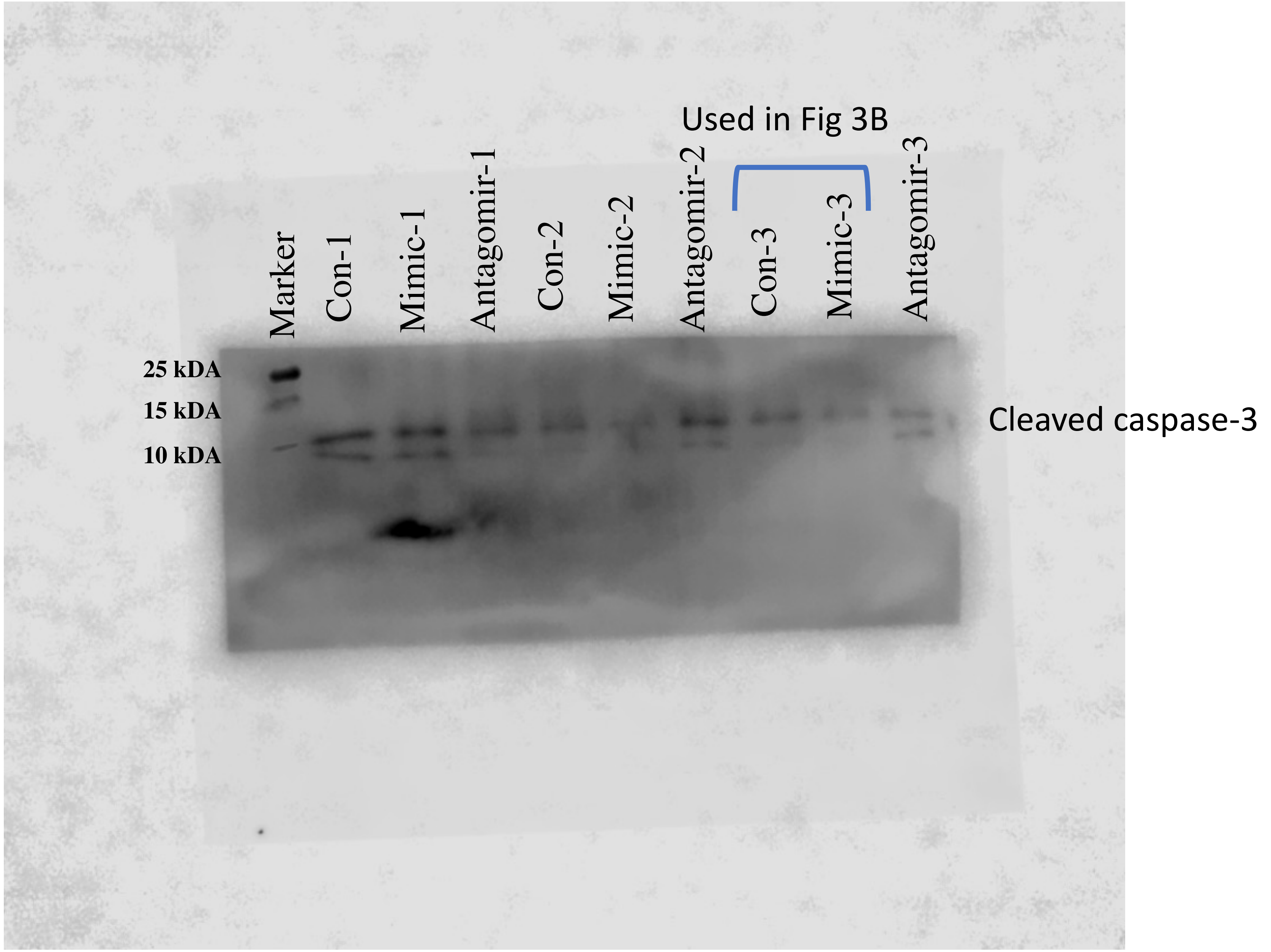


Bands were visualized with ECL substrate using chemiluminescence channel in LiCor Fc Odyssey system.

Membrane 8

Membrane 8 was cut to incubate different antibodies on the same day. Order of antibody incubation: p-eNOS, GAPDH, P21, then eNOS and PDIA4

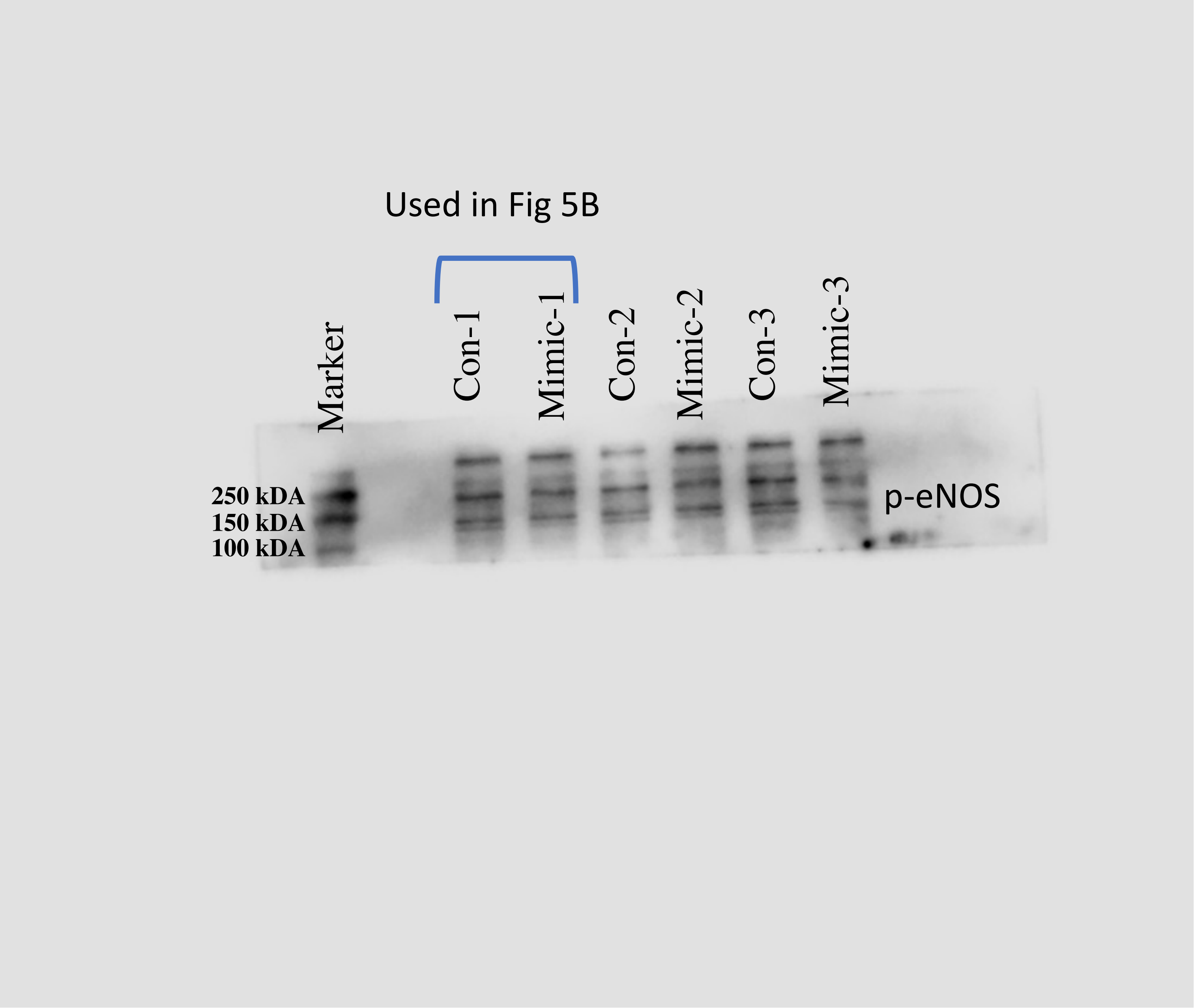

Membrane 8

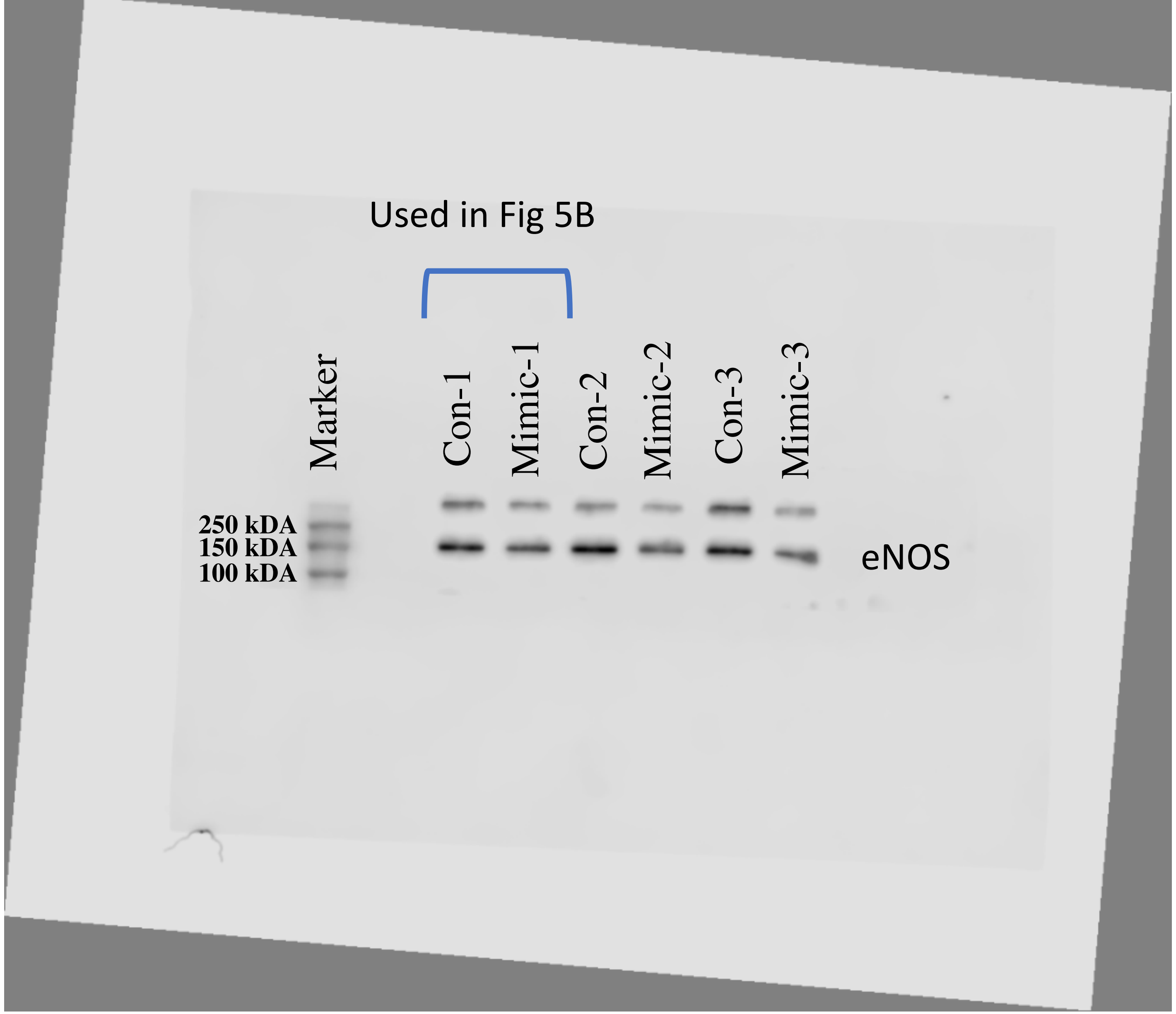
Used in Fig 5B
Marker
Con-1
Mimic-1
Con-2
Mimic-2
Con-3
Mimic-3
250 kDA
150 kDA
100 kDA
eNOS

Membrane 8

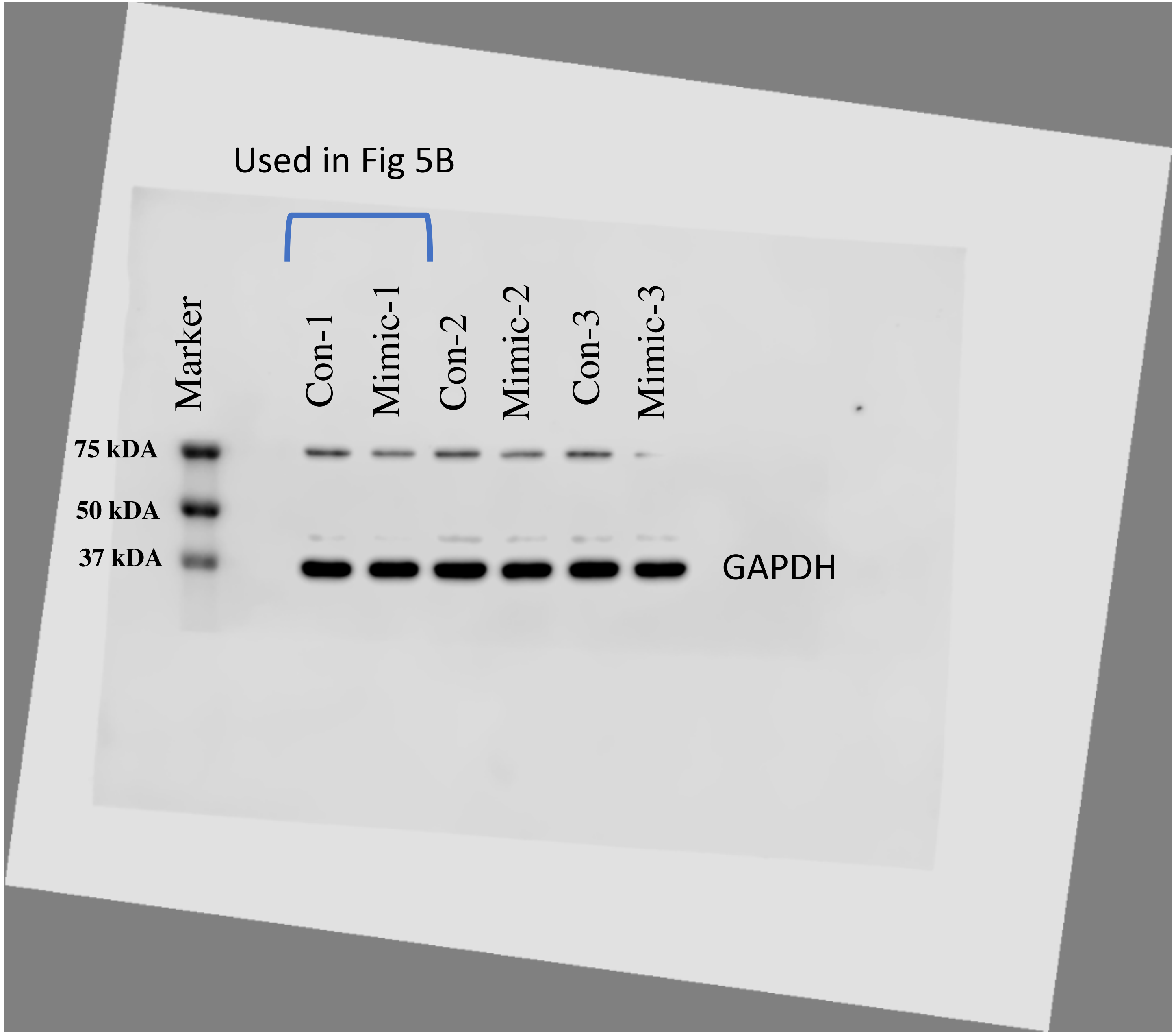

Used in Fig 5B
Marker
Con-1
Mimic-1
Con-2
Mimic-2
Con-3
Mimic-3
75 kDA
50 kDA
37 kDA
GAPDH

Membrane 8

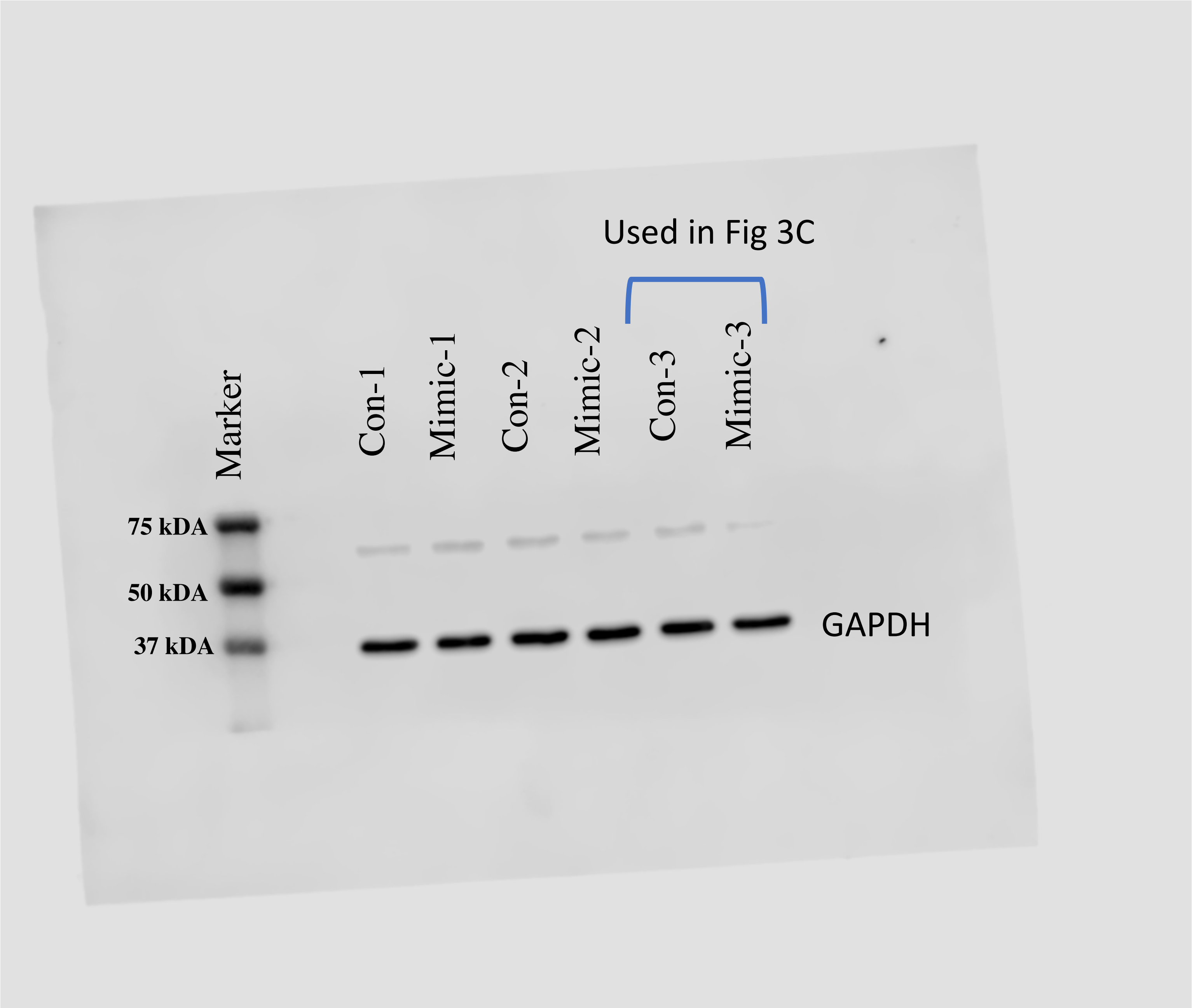


Bands were visualized with ECL substrate using chemiluminescence channel in LiCor Fc Odyssey system.

Membrane 8

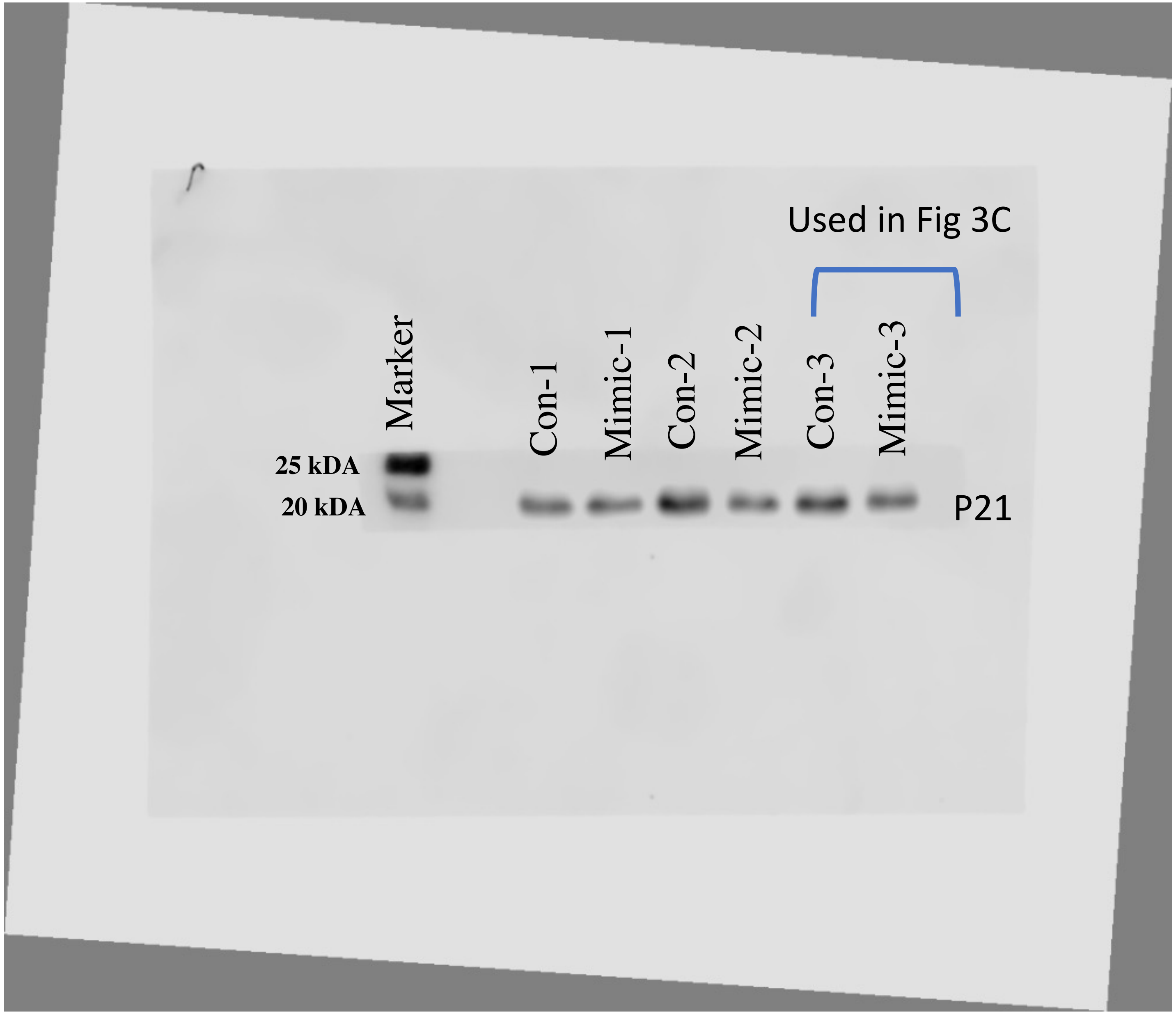
Used in Fig 3C
Marker
Con-1
Mimic-1
Con-2
Mimic-2
Con-3
Mimic-3
25 kDA
20 kDA
P21

Membrane 8

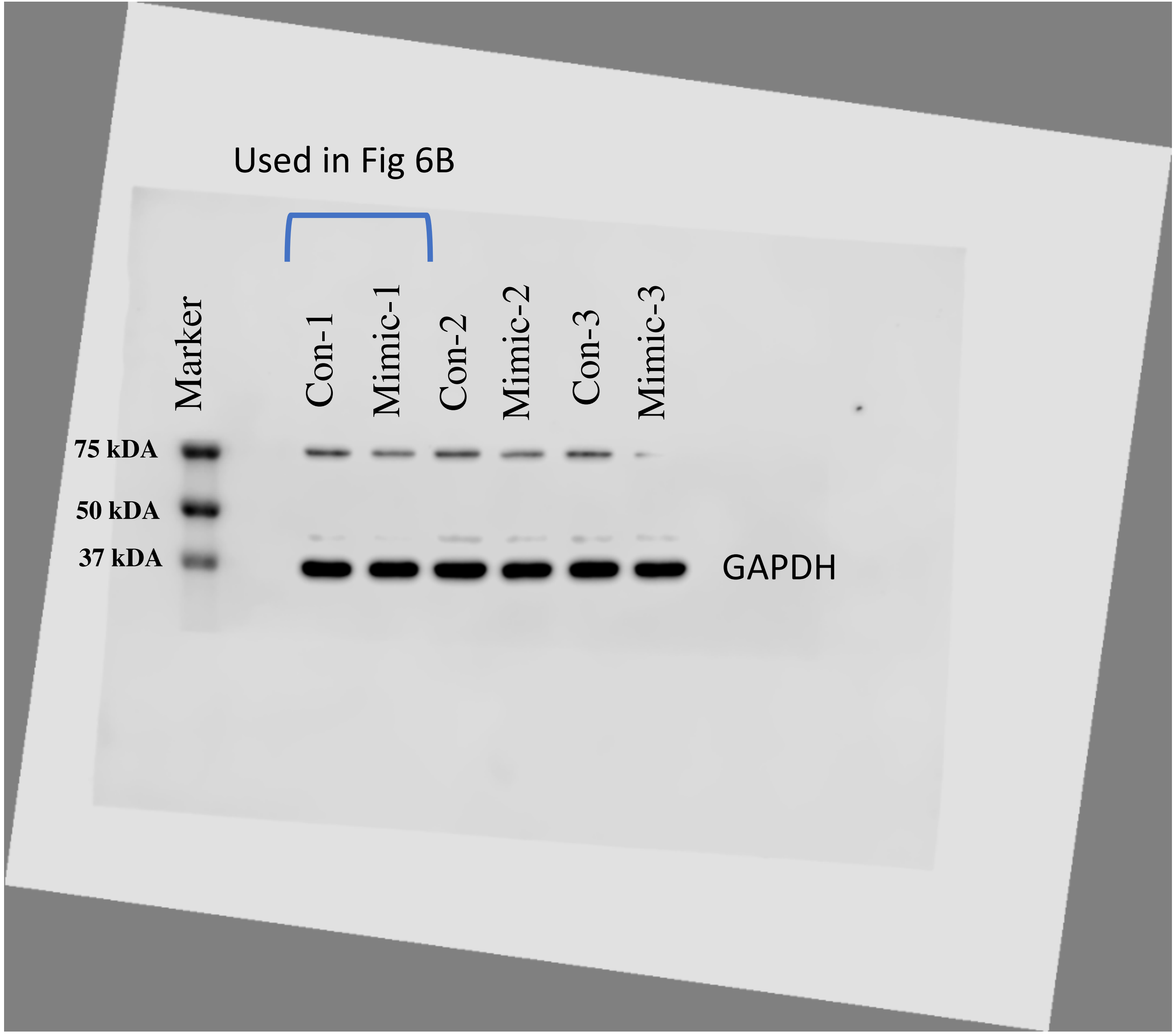
Used in Fig 6B
Marker
Con-1
Mimic-1
Con-2
Mimic-2
Con-3
Mimic-3
75 kDA
50 kDA
37 kDA
GAPDH

Membrane 10

Order of antibody incubation: p-eNOS then eNOS then PDIA4 then GAPDH

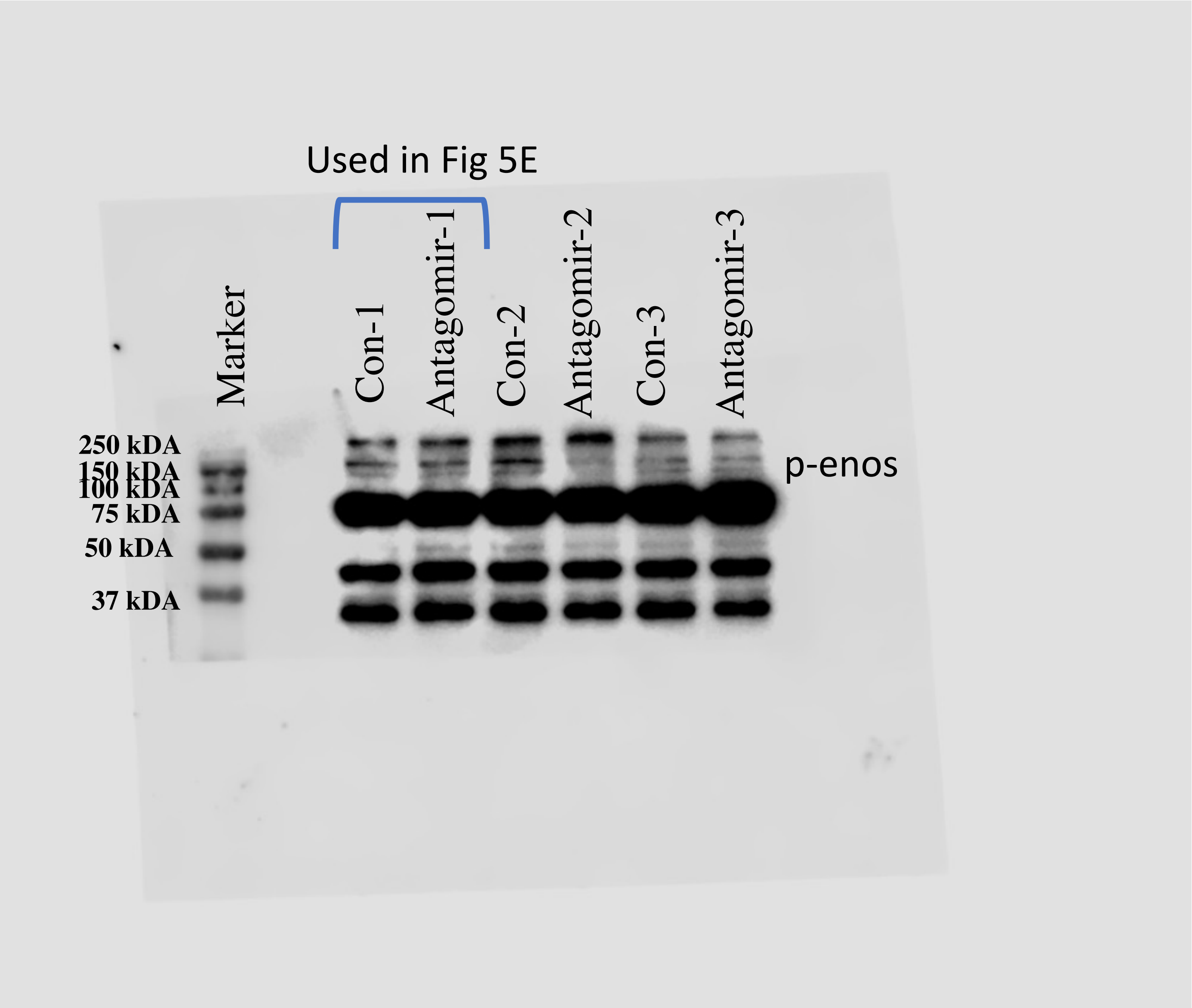

Membrane 10

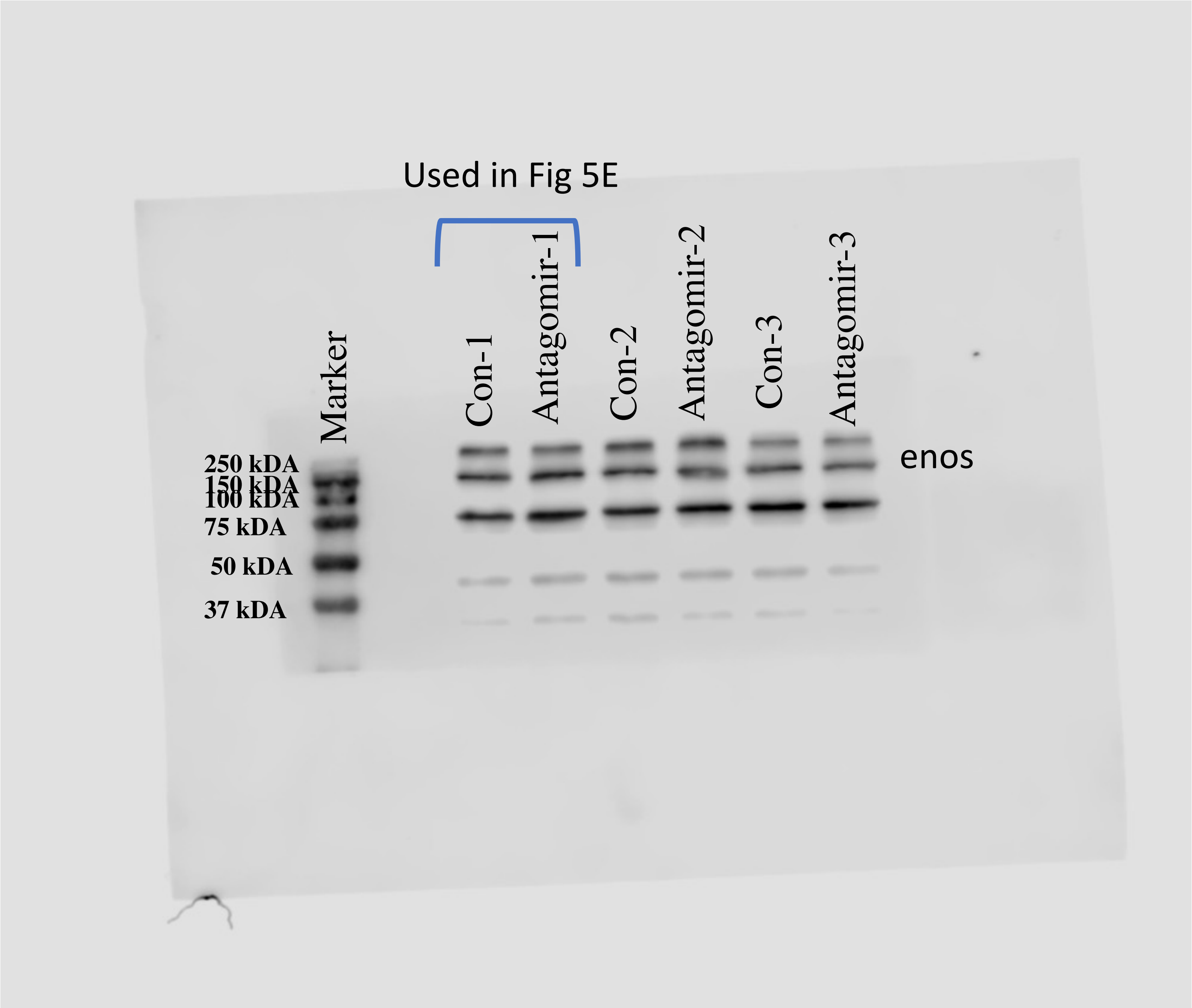
Used in Fig 5E
Marker
Con-1
Antagomir-1
Con-2
Antagomir-2
Con-3
Antagomir-3
250 kDA
150 kDA
100 kDA
75 kDA
50 kDA
37 kDA
enos

Membrane 10

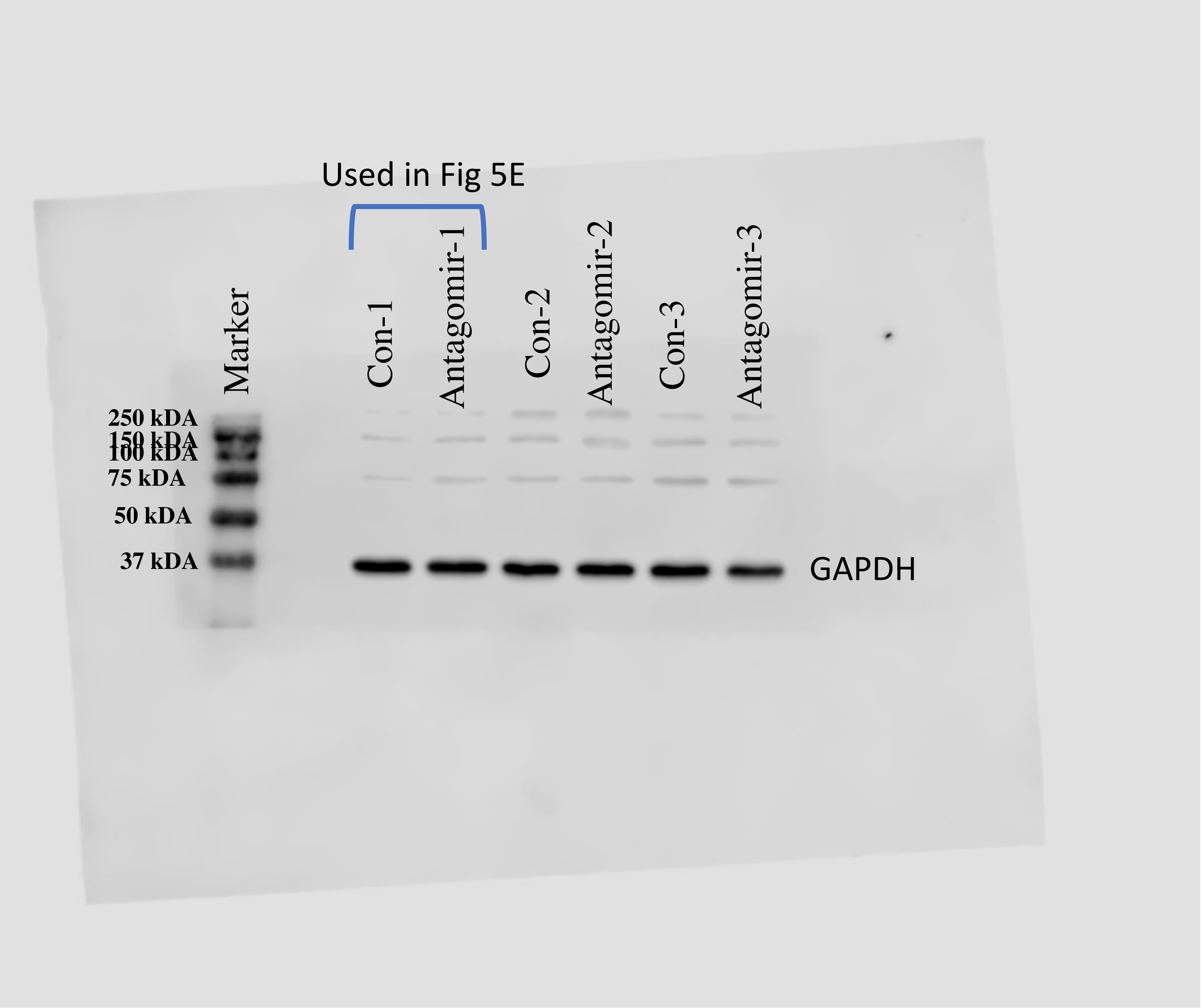
Used in Fig 5E
Marker
Con-1
Antagomir-1
Con-2
Antagomir-2
Con-3
Antagomir-3
250 kDA
150 kDA
100 kDA
75 kDA
50 kDA
37 kDA
GAPDH

Membrane 10

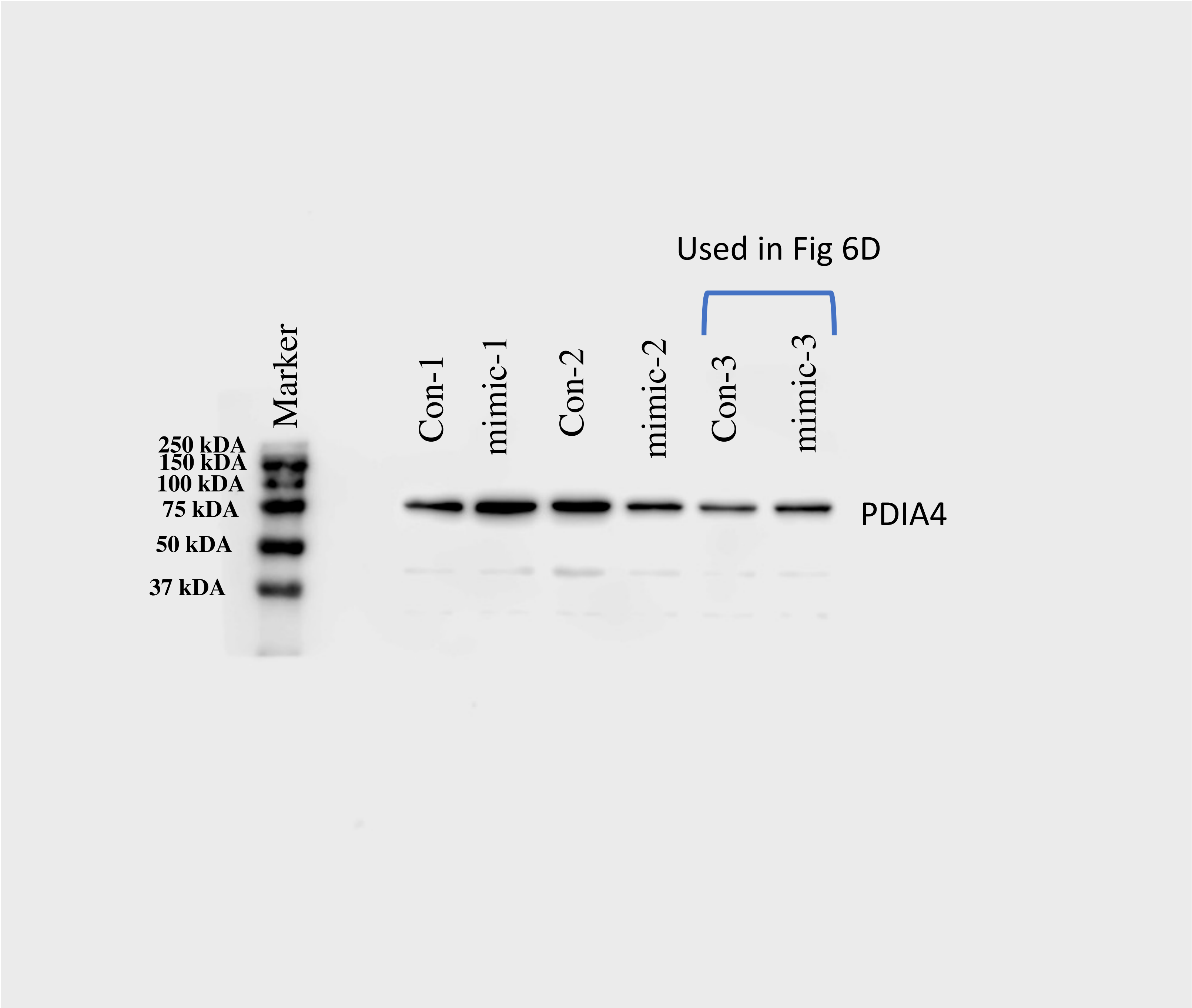
Used in Fig 6D
Marker
Con-1
mimic-1
Con-2
mimic-2
Con-3
mimic-3
250 kDA
150 kDA
100 kDA
75 kDA
50 kDA
37 kDA
PDIA4

Membrane 10

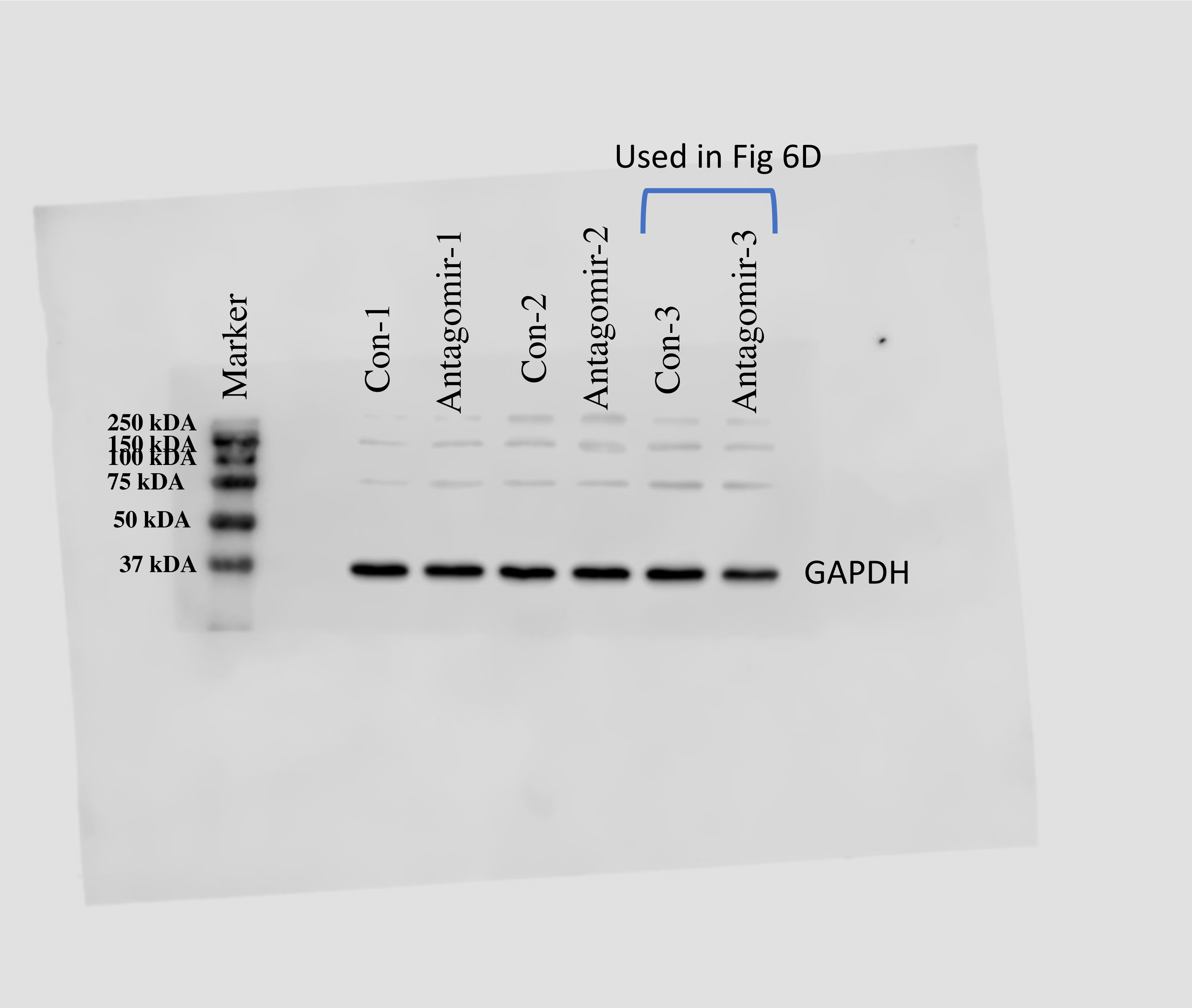

Used in Fig 6D
Marker
Con-1
Antagomir-1
Con-2
Antagomir-2
Con-3
Antagomir-3
250 kDA
150 kDA
100 kDA
75 kDA
50 kDA
37 kDA
GAPDH

Membrane 11

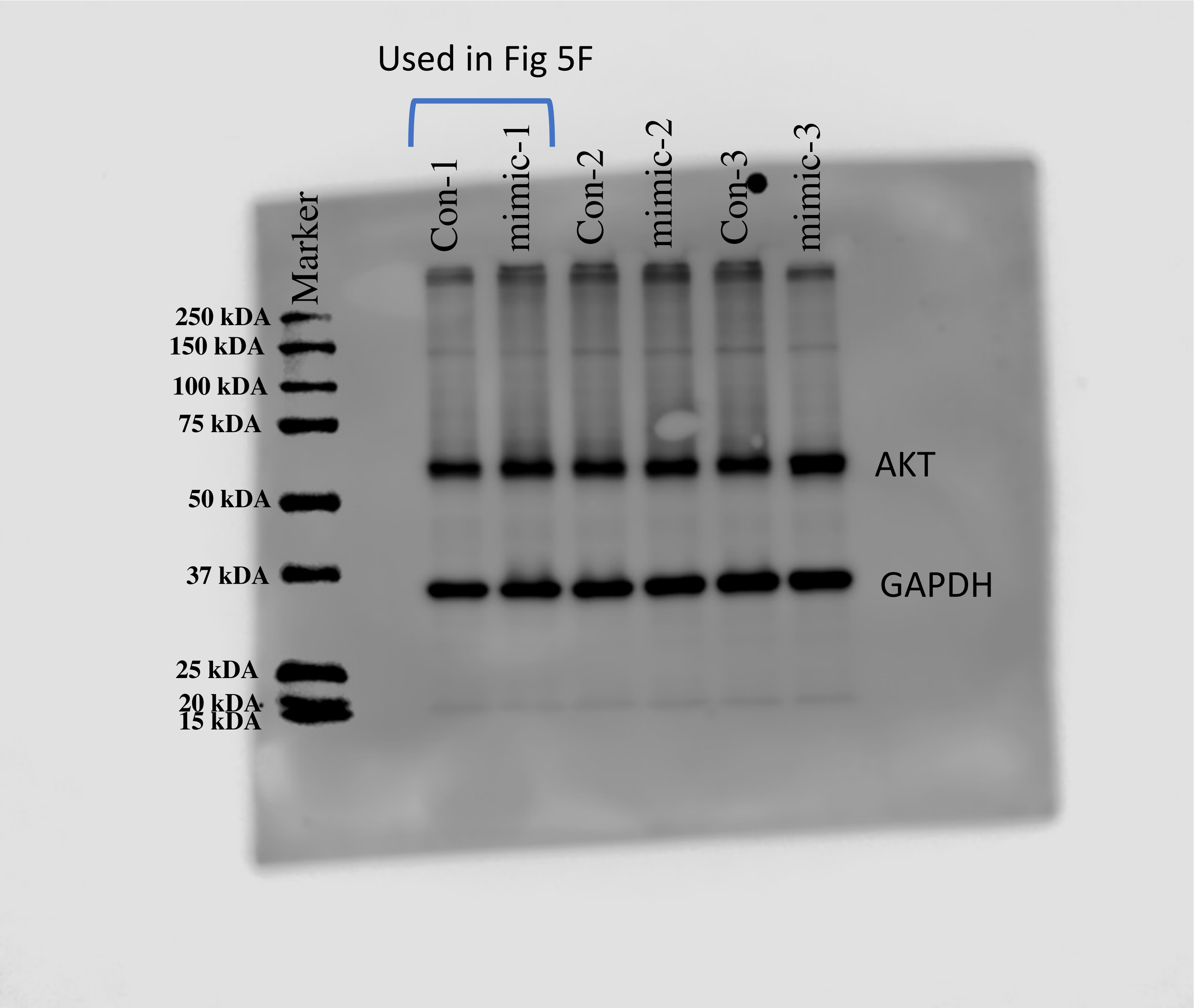


Western blot was first probed with AKT then GAPDH antibody.

Membrane 12

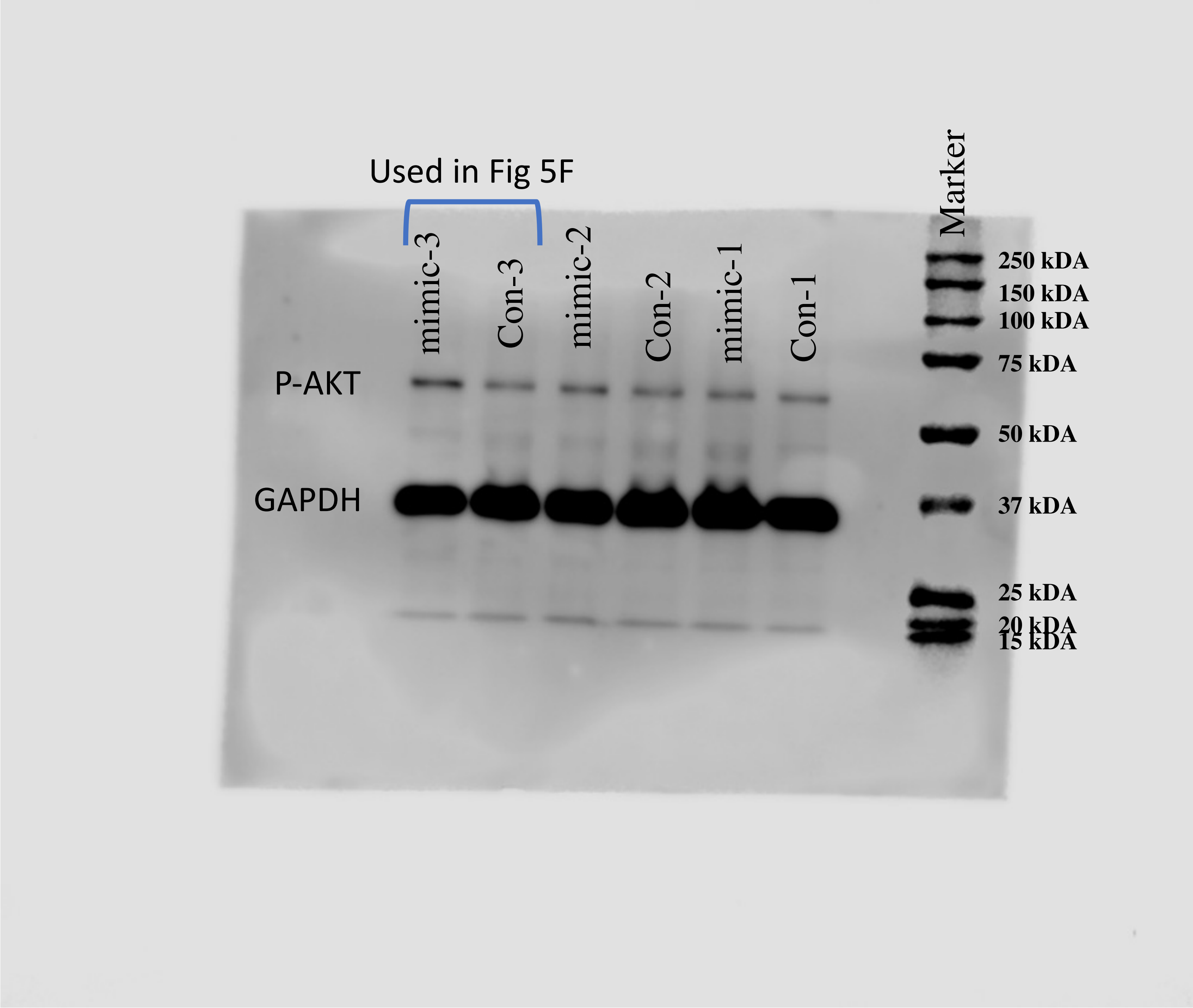


Western blot was first probed with p-AKT then GAPDH antibody.
